# Contradictory Phylogenetic Signals in the Laurasiatheria Anomaly Zone

**DOI:** 10.3390/genes13050766

**Published:** 2022-04-26

**Authors:** Liliya Doronina, Graham M. Hughes, Diana Moreno-Santillan, Colleen Lawless, Tadhg Lonergan, Louise Ryan, David Jebb, Bogdan M. Kirilenko, Jennifer M. Korstian, Liliana M. Dávalos, Sonja C. Vernes, Eugene W. Myers, Emma C. Teeling, Michael Hiller, Lars S. Jermiin, Jürgen Schmitz, Mark S. Springer, David A. Ray

**Affiliations:** 1 Institute of Experimental Pathology, ZMBE, University of Münster, 48149 Münster, Germany; jueschm@uni-muenster.de; 2 School of Biology and Environmental Science, University College Dublin, Belfield, D04 V1W8 Dublin, Ireland; colleen.lawless@ucdconnect.ie (C.L.); tadhg.lonergan@ucdconnect.ie (T.L.); louise.ryan1@ucdconnect.ie (L.R.); emma.teeling@ucd.ie (E.C.T.); lars.jermiin@anu.edu.au (L.S.J.); 3 Department of Biological Sciences, Texas Tech University, Lubbock, TX 79409, USA; diana.moreno@berkeley.edu (D.M.-S.); j.korstian@ttu.edu (J.M.K.); 4 Department of Integrative Biology, University of California, Berkeley, CA 92697, USA; 5 Max Planck Institute of Molecular Cell Biology and Genetics, 01307 Dresden, Germany; jebbdaithi@gmail.com (D.J.); myers@mpi-cbg.de (E.W.M.); 6 Max Planck Institute for the Physics of Complex Systems, 01187 Dresden, Germany; 7 Center for Systems Biology Dresden, 01307 Dresden, Germany; 8 LOEWE Centre for Translational Biodiversity Genomics, 60325 Frankfurt, Germany; bogdan.kirilenko@senckenberg.de (B.M.K.); michael.hiller@senckenberg.de (M.H.); 9 Senckenberg Research Institute, 60325 Frankfurt, Germany; 10 Faculty of Biosciences, Goethe-University, 60438 Frankfurt, Germany; 11 Department of Ecology and Evolution and Consortium for Inter—Disciplinary Environmental Research, Stony Brook University, Stony Brook, NY 11794, USA; liliana.davalos@stonybrook.edu; 12 School of Biology, The University of St Andrews, St Andrews KY16 9ST, UK; scv1@st-andrews.ac.uk; 13 Neurogenetics of Vocal Communication Group, Max Planck Institute for Psycholinguistics, 6525 Nijmegen, The Netherlands; 14 Faculty of Computer Science, Technical University Dresden, 01307 Dresden, Germany; 15 The Okinawa Institute of Science and Technology, Okinawa 904-0495, Japan; 16 Research School of Biology, Australian National University, Canberra, ACT 2601, Australia; 17 Earth Institute, University College Dublin, D04 V1W8 Dublin, Ireland; 18 Department of Evolution, Ecology and Organismal Biology, University of California, Riverside, CA 92521, USA; springer@ucr.edu

**Keywords:** retrophylogenomics, exon concatenation, exon coalescence, Laurasiatheria, Scrotifera, anomaly zone

## Abstract

Relationships among laurasiatherian clades represent one of the most highly disputed topics in mammalian phylogeny. In this study, we attempt to disentangle laurasiatherian interordinal relationships using two independent genome-level approaches: (1) quantifying retrotransposon presence/absence patterns, and (2) comparisons of exon datasets at the levels of nucleotides and amino acids. The two approaches revealed contradictory phylogenetic signals, possibly due to a high level of ancestral incomplete lineage sorting. The positions of Eulipotyphla and Chiroptera as the first and second earliest divergences were consistent across the approaches. However, the phylogenetic relationships of Perissodactyla, Cetartiodactyla, and Ferae, were contradictory. While retrotransposon insertion analyses suggest a clade with Cetartiodactyla and Ferae, the exon dataset favoured Cetartiodactyla and Perissodactyla. Future analyses of hitherto unsampled laurasiatherian lineages and synergistic analyses of retrotransposon insertions, exon and conserved intron/intergenic sequences might unravel the conflicting patterns of relationships in this major mammalian clade.

## 1. Introduction

Insights into organismal phylogeny are crucial for most modern biological studies. In the genomics era, the availability of high-quality genome assemblies and innovative phylogenomic methods provide the opportunity to resolve many longstanding questions. However, modern phylogenomic sequence analyses with both concatenation and coalescence methods can be undermined when model assumptions are violated, and are especially challenging in the case of ancient, rapid radiations that reside in the anomaly zone [1]. Phylogenetic reconstructions in such anomaly zones, which occur when consecutive branch lengths (in coalescent units) on the species tree are very short [1], vary substantially depending on taxon sampling, data sets, marker systems, and methods applied. With a short duration between successive speciation events, faster than necessary for marker fixation (<2 million years for primates [2]), polymorphic states are inherited by descendant lineages followed by random fixation. This may lead to a random rather than phylogenetically consistent fixation of alleles—a phenomenon known as incomplete lineage sorting (ILS). ILS is an attendant problem for diversification in many groups including mammals [3,4,5], birds [6,7], and other vertebrates [8,9]. The shorter the period between speciation events, the weaker the historical signal and the louder the phylogenetic noise. When ILS exceeds a certain level and overlays the historical signal, relationships cannot be accurately reconstructed and may yield a hard polytomy. Such a scenario, for example, was suggested for neoavian birds [10].

A prominent case of an anomaly zone is the early diversification of ordinal lineages in the superorder Laurasiatheria. This superorder includes six orders: Eulipotyphla (moles, shrews), Chiroptera (bats), Perissodactyla (horses, rhinos), Cetartiodactyla (pigs, cows, whales), Carnivora (dogs, cats), and Pholidota (pangolins). Eulipotyphla occupies the widely accepted basal position in laurasiatherians. The remaining five orders form the clade called Scrotifera, but include only one agreed phylogenetic relationship, the monophyletic Ferae (Carnivora + Pholidota) [3,11].

The most recent common ancestor of Scrotifera diversified rapidly into four lineages (Chiroptera, Perissodactyla, Cetartiodactyla, Ferae) in the Late Cretaceous [12,13,14]. Foley et al.’s [14] timetree estimates suggest that scrotiferan cladogenesis into four lineages began ~78 million years ago and required less than two million years [14]. This episode of rapid speciation is also characterized by a high level of ILS. Hence, the scrotiferan diversification represents one of the most challenging problems in higher-level mammalian phylogenetics.

Multiple large-scale sequence datasets have been applied to this problem, including protein-coding regions, introns, and ultra-conserved elements (UCEs) [15,16,17,18]. These studies have recovered well-supported but sometimes contradictory phylogenetic relationships even in cases where analyses of the same dataset were performed with different phylogenetic methods. However, such inconsistencies across data types and phylogenetic methods are not uncommon for rapidly radiating groups (e.g., [19]) and are even expected when species trees are in the anomaly zone. To illustrate these inconsistencies for Laurasiatheria, Chen et al.’s [17] genome-scale intron analysis (introns from 3638 genes) revealed Chiroptera–Perissodactyla and Cetartiodactyla–Carnivora sister group relationships. By contrast, Chen et al.’s [17] analyses with protein-coding sequences (10,259 genes) recovered an alternative position for Perissodactyla as the sister group to Cetartiodactyla–Carnivora, albeit with weak bootstrap support. Chen et al. [17] concluded that introns outperformed protein-coding sequences, but a complication with this conclusion is that Chen et al.’s [17] protein-coding sequences are compromised by large-scale homology problems, including introns that are aligned to exons [20]. By contrast with Chen et al. [17], most of Esselstyn et al.’s [21] analyses of UCEs (3787 loci) recovered a basal split in Scrotifera between Chiroptera and Fereuungulata (Ferae + Perissodactyla + Cetartiodactyla) as well as a sister-group relationship between Perissodactyla and Cetartiodactyla (Euungulata). Liu et al. [22] also recovered a basal split between Chiroptera and other scrotiferans, as well as a monophyletic Euungulata, based on summary coalescence analyses of 4388 protein-coding genes with STAR and NJst. By contrast, some of Liu et al.’s [22] concatenation analyses placed Chiroptera as the sister group to Cetartiodactyla. A caveat of Liu et al.’s [22] analyses is that they were also influenced by large-scale homology problems [23]. Du et al. [24] employed trimAL and two different filtering protocols to create updated versions of Liu et al.’s [22] dataset that included 5162 loci. These authors inferred species trees from these datasets using both concatenation and summary coalescence methods with different substitution models. Two of the coalescence methods (STAR, NJst) consistently supported a basal split between Chiroptera and Fereuungulata and a sister-group relationship between Cetartiodactyla and Perissodactyla. ASTRAL also recovered a basal split between Chiroptera and Fereuungulata, but by contrast with the other two coalescence methods, always recovered a sister-group relationship between Perissodactyla and Cetartiodactyla + Ferae group. Concatenation analyses sometimes supported Fereuungulata and Euungulata as monophyletic clades, but more commonly recovered Chiroptera and Perissodactyla as sister taxa. Jebb et al. [25] analysed both protein-coding genes (12,931 loci) and conserved noncoding elements (CNEs, 10,857 loci) with concatenation and recovered Fereuungulata with both data sets. Within Fereuungulata, concatenation analysis of the protein-coding dataset supported Euungulata, whereas analysis of the CNEs supported Zoomata (Ferae + Perissodactyla). Concatenation was also performed with a smaller data set that consisted of 488 protein-coding loci that displayed an optimal fit to the model of sequence evolution. Concatenation recovered Euungulata and a sister-group relationship between Ferae and Chiroptera, although the latter was only weakly supported. SVDquartets analysis of this dataset supported Fereuungulata and Euungulata. The most recent study of scrotiferan phylogeny [18] analysed four different phylogenomic data sets (proteins and their corresponding protein-coding sequences (4186 loci), introns (1210 loci), UCEs (1246 loci)) with both concatenation and summary coalescence methods. All of the analyses resulted in congruent results that support the monophyly of both Fereuungulata and Euungulata [18].

It is well known that concatenation ignores the effects of ILS and is expected to fail when there has been rapid radiation and the species tree is in the anomaly zone [1]. Summary coalescence methods explicitly address the effects of ILS and have the potential to shed light on the phylogeny of rapidly diversifying groups (for review, see [26]). However, the application of different ILS-aware methods to scrotiferan datasets, as summarized above, has resulted in mutually exclusive tree topologies [26]. The probable reason for these discrepancies is violations of the assumptions that underlie summary coalescence methods. The first important assumption is that all gene tree heterogeneity results from ILS, but for empirical data sets, ILS is dwarfed by other sources of gene tree heterogeneity [27,28]. A second assumption is that there is free recombination between coalescence genes (c-genes), but not within c-genes [29]. Scornavacca and Galtier [28] analysed a phylogenomic dataset of protein-coding sequences that included 39 placental mammals and concluded that different exons of the same protein-coding gene do not share the same genealogy and therefore are not part of the same c-gene. These results imply that complete protein-coding sequences, which have been employed in numerous studies with summary coalescence methods (e.g., [17,22,24,30]), are inappropriate units for the application of summary coalescence methods [31]. At the same time, Scornavacca and Galtier [28] suggested that individual exons may be appropriate genomic units for gene-tree-based phylogenomic analyses because the two halves of the same exon show a positively correlated phylogenetic signal.

Besides ILS, phylogenetic noise is produced by homoplasy (independent occurrence of identical character states in unrelated lineages), which might be especially problematic in critical phylogenetic reconstructions. Here, a marker system with a low homoplasy level can help overcome the problem. Retrotransposons, which are mobile genomic elements, have become increasingly popular in phylogenomic studies, especially in addressing the phylogeny of clades that have undergone rapid radiations [32,33,34]. Spreading across genomes using a “copy-and-paste” mechanism, retrotransposons, once inserted randomly in a genomic locus and fixed in the population, transmit to all descendent lineages. Thus, the presence of a retrotransposon in a specific genomic locus reveals the lineages’ relatedness, whereas all distant relatives show “absence.” Because cases of parallel insertions of retrotransposons at the exact genomic location in different species or their precise deletions are extremely rare, they provide virtually homoplasy-free data sources [33,35]. When retrotransposon insertions support conflicting topologies, the most likely cause is incomplete ancestral lineage sorting rather than homoplasy [2,6].

Retrophylogenomic approaches were previously applied to investigate the phylogenetic relationships in Scrotifera [36]. The screening of thousands of retrotransposons in Chiroptera, Perissodactyla, Cetartiodactyla, and Carnivora revealed 162 markers that supported a network of all possible interordinal affiliations. The Pegasoferae hypothesis (Chiroptera–Perissodactyla–Ferae), which was originally proposed based on a relatively small retrotransposon presence/absence dataset [37], received insignificant support in this study. Within the scrotiferan network, retrotransposon presence/absence patterns of Doronina et al. [36] identified a basal position for Chiroptera and a Cetartiodactyla–Ferae group affiliation named Cetartioferae. However, support was only moderate (neighbor-net and most parsimonious tree reconstructions). Perissodactyla shares 14 retrotransposons with Cetartioferae but also 11 retrotransposons with Chiroptera [36]. The 4-lineage analysis designed for retrotransposon insertions (4-LIN [38]) explained such ambivalence of Perissodactyla by the hybridization of ancestral chiropterans with the ancestors of either Carnivora or a Cetartiodactyla–Carnivora precursor [36]. However, a Quartet-Asymmetry test [34] did not confirm hybridization and suggested that ILS alone explains the marker distribution in Laurasiatheria.

It was proposed that retrotransposon presence/absence data might have advantages over classical sequence analyses in multispecies coalescent (MSC) methods due to their virtually homoplasy-free nature, the absence of intra-locus recombination, and relaxed selection (e.g., [26,34]). A reanalysis of Doronina et al.’s [36] data with ILS-aware MSC methods (ASTRAL_BP and SDPquartets analyses [34]) recovered Cetartioferae, albeit with low support. The ASTRAL_BP analysis also recovered consecutive short branch lengths, in coalescent units, that position the Laurasiatheria polytomy in the anomaly zone [34].

The use of c-genes has been proposed as a means of overcoming problems impacting phylogenetic inference in addition to using retrotransposon data. As detailed above, c-genes represent regions of a chromosome that have not undergone recombination, and some may maintain an accurate record of the evolutionary history of a clade provided they are large enough to contain phylogenetic signals [31]. An additional consideration is that c-genes become smaller with increased taxon sampling because of the recombination ratchet [31]. While coding gene sequence data are a common source of information in molecular phylogenetic analyses, identifying target genes for which no component exons (or intervening introns) have undergone recombination remains challenging. One solution is to treat each exon as an independent c-gene, provided that orthologous exons can be identified with certainty. Doing so, however, entails analysing many short alignments of data, implying that the sample size may be too small for each alignment to gain a consistent phylogenetic estimate from each exon.

**Figure 1:**
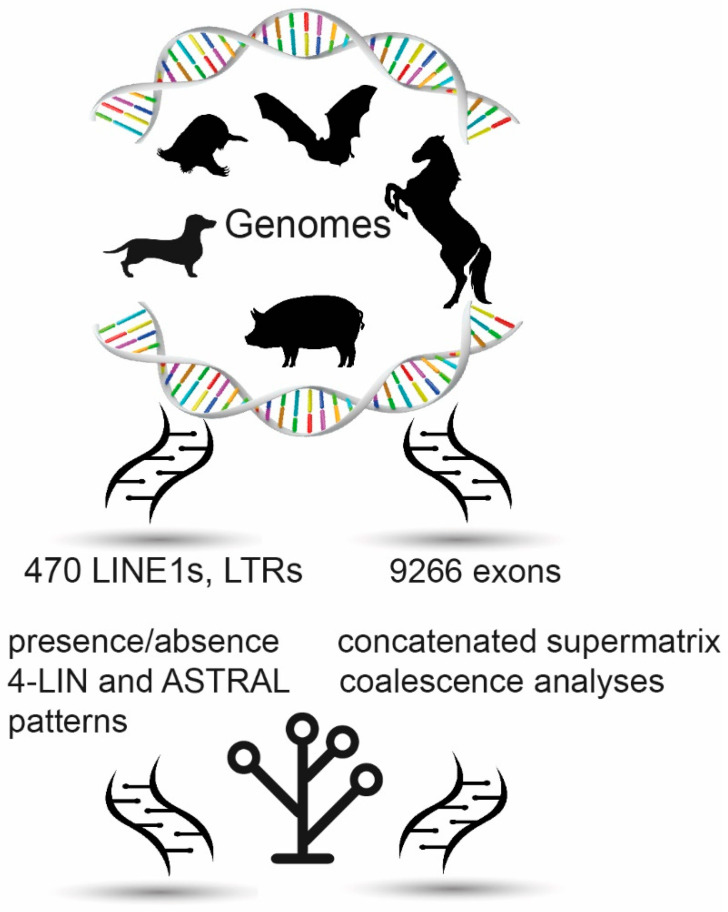
Schematic representation of the applied phylogenetic approaches. Retrotransposon analyses of 470 diagnostic LINE1 and LTR presence/absence patterns (**left**) and exon sequence for concatenated and coalescence analyses (**right**).

Both sequence- and retrotransposon-based studies are strongly dependent on the quality of genome assemblies, and the investigation of such anomaly zones as Laurasiatheria requires a large-scale analysis of high-quality genomic data. The recently published reference-quality genomes of several bat species [25] provide a new perspective to untangle the evolutionary history of Laurasiatheria. Here, we leverage these new genomes and analyse both retrotransposon presence/absence patterns and ultra-high-quality exon datasets of all five laurasiatherian lineages—Eulipotyphla, Chiroptera, Perissodactyla, Cetartiodactyla, and Ferae (Figure 1)—to provide new insights into laurasiatherian phylogenetic relationships.

## 2. Materials and Methods

### 2.1. Retrotransposon Analyses

#### 2.1.1. Genome Assemblies and 2-Way Alignments

We performed the retrotransposon presence/absence screening with the following laurasiatherian genomes: star-nosed mole (*Condylura cristata*) for Eulipotyphla, greater mouse-eared bat (*Myotis myotis*) for Chiroptera, the domestic horse (*Equus ferus caballus*) for Perissodactyla, pig (*Sus scrofa*) for Cetartiodactyla, and domestic dog (*Canis lupus familiaris*) for Ferae. With these, we generated pairwise 2-way whole-genome alignments (target/query): (1) bat/dog, (2) bat/pig, (3) bat/horse, (4) bat/mole, (5) dog/bat, (6) dog/pig, (7) dog/horse, (8) dog/mole, (9) pig/bat, (10) pig/dog, (11) pig/horse, (12) pig/mole, (13) horse/bat, (14) horse/dog, (15) horse/pig, and (16) horse/mole. Briefly, we used LASTZ (version 1.04.03) [39] with sensitive alignment parameters (K = 2400, L = 3000, Y = 9400, H = 2000 and the LASTZ default scoring matrix) to obtain local alignments. AxtChain [40] (default parameters except linearGap = loose) was used to compute co-linear alignment chains, RepeatFiller [41] (default parameters) to capture previously missed alignments between repetitive regions and chainCleaner [42] (default parameters except minBrokenChainScore = 75000 and -doPairs) to improve alignment specificity.

#### 2.1.2. Screening for Phylogenetically Informative Retrotransposons

We identified the genomic coordinates of retrotransposons in the genomes of the star-nosed mole, greater mouse-eared bat, horse, pig, and dog using a local version of the RepeatMasker (version 4.0.7) (http://www.repeatmasker.org/RepeatMasker/ (accessed on 28 March 2022)). We used the recently developed 2-n-way tool [43] to trace the presence or absence of retrotransposons of target species at orthologous genomic positions in query species. Pairwise 2-way whole-genome alignments were uploaded to the n-way module. Using standard n-way settings, we then performed an exhaustive, multi-directional screening for phylogenetically informative retrotransposon presence/absence patterns. To increase the accuracy of the analyses, we applied an n-way-embedded, MUSCLE-based optimization. We focused on two retrotransposon groups, LINE1s (3′-truncation < 50 nt) and LTRs (5′- and 3′-truncations ≤ 20 nt), which were verifiably active during the time of laurasiatherian diversification [36]. To evaluate all the potential tree topologies of the investigated laurasiatherian lineages, we searched for presence/absence patterns for all 25 possible interordinal affiliations (e.g., exclusive presence in bat + dog, bat + dog + pig, see Supplementary Table S1). From the n-way, we sampled all alignments of loci (diagnostic retrotransposons with 500-nt flanks) with perfect presence/absence patterns (distinct n-way (+) or (−) assignments). Each of these was then supplemented with a second species for every order (Supplementary Table S2) from genome assemblies of laurasiatherians available at the National Center for Biotechnology Information (NCBI, https://ncbi.nlm.nih.gov (accessed on 28 March 2022)), and UCSC Genome Browser Database (https://genome.ucsc.edu (accessed on 28 March 2022)). The second species helped reduce species-specific signals resulting from occasional instances of homoplasy [33]. As there is already strong evidence for the monophyly of Ferae (Carnivora + Pholidota) (e.g., [11,18,25,36,44]), we omitted pangolin from the initial computational screenings. However, we subsequently added sequence information of pangolins to the alignments. We also complemented all alignments with a representative of Euarchontoglires for outgroup comparison (Supplementary Table S2). Every individual alignment was manually analysed to verify orthology and presence/absence patterns. We accepted as phylogenetically informative markers only retrotransposon insertions at orthologous positions flanked by target site duplications (TSDs) shifted in their genomic location less than three nucleotides between different species, representing the same element type in the same orientation, and exhibiting a clear absence state in the outgroup. The alignments of retrotransposon markers are presented in Supplementary File S1. The table with the number of retrotransposon markers found for all possible laurasiatherian order affiliations and the presence/absence table of retrotransposon markers are presented as Supplementary Tables S1 and S2, respectively.

#### 2.1.3. Phylogenetic Reconstructions

We built two presence/absence (1/0) data matrices for retrotransposon markers: one with markers found in the present screening and the other also including all the non-overlapping markers from Doronina et al. [36] (Supplementary File S2). The two matrices were analysed in SplitsTree (version 4.13.1, [45]) using neighbor-net analysis with standard settings and 1000 bootstrap replicates. SplitsTree allows for the visualization of data conflicts within a phylogenetic network. We also applied Dollo parsimony as implemented in the Dollop program in PHYLIP (version 3.695, Dollo and Polymorphism Parsimony [46]) to infer the most parsimonious tree with standard parameters and randomized input order of species (seven times to jumble, random seed “13131”). Dollop admits only one forward change to gain a character and minimizes the number of reversions to explain presence/absence patterns. Dollo parsimony outperformed other variants of parsimony in analyses of simulated retroelement datasets [47]. MrBayes v3.2.5 was applied for a Bayesian inference using the Standard Discrete Model (binary, i.e., 1/0 character states) and ctype (irreversible, originally used for morphological data) for all characters, e.g., ctype irreversible: 1–470, with datatype = standard, mcmc ngen = 20,000, samplefreq = 100, printfreq = 100, and diagnfreq = 1000 [48]. We also applied three ILS-aware MSC methods: ASTRAL_BP, ASTRID_BP, and SDPquartets [34,47]. Bootstrap analyses with the coalescence methods were performed with 1000 pseudoreplications.

To further identify phylogenetic relationships within Scrotifera, we built two additional datasets with and without the data of Doronina et al. [36] and omitted Eulipotyphla to apply the 4-lineage statistical test for diagnostic presence/absence markers (4-LIN, reverse algorithm, empirical distribution, [38]). This test is designed specifically for the presence/absence data of four-lineage phylogenetic relationships. 4-LIN also considers multiple hybridization scenarios and provides a tree/net with the highest log-likelihood values using an embedded χ^2^ test for the evaluation. To further analyse possible hybridization, we applied a Quartet-Asymmetry test [34] using the 470-dataset.

### 2.2. Coding Exon Data Analyses

#### 2.2.1. Generating Mammalian Exon Alignments

We generated sequence alignments for 9266 exons across 3911 genes and 47 mammal species (available at Figshare, https://figshare.com/s/b0bca1ca0f8328ec993a (accessed on 28 March 2022); most of the species used by Jebb et al. [25] were taken in the present analyses (Supplementary Figure S3); for the cow and cat, we used the updated bosTau9 and felCat9 assemblies, respectively). These high-quality exon sequence alignments were generated by filtering TOGA (https://github.com/hillerlab/TOGA/ (accessed on 28 March 2022)) annotations for intact exons of 1:1 orthologous genes that have an intact reading frame and that satisfy a minimum coverage of phylogeny-important species. We required here that each exon align to at least two primates, at least one representative of Rodentia and Lagomorpha, at least two representatives of Afrotheria and Xenarthra, one Pholidota, three Cetartiodactyla, three Carnivora, one Perissodactyla, five Chiroptera, and one Eulipotyphla. Split codons were trimmed, resulting in individual exon sequences that are a multiple of 3 bp. The median and average length of the exons is 114 and 137 bp. The exons ranged in length from 30–2271 bp. Both nucleotide and translated amino acid sequences were used in all downstream exon analyses (Supplementary Figure S1). The use of individual exons, rather than complete protein-coding sequences, greatly reduces the likelihood that these markers violate the ‘no intralocus recombination’ assumption of summary coalescence methods [28].

#### 2.2.2. Inference of Best-Fit Models of Sequence Evolution and Exon Tree

We used ModelFinder [49] implemented in IQTREE2 [50] to determine the best-fit model of sequence evolution (SE) per gene per data type. For DNA, all nucleotide substitution models were explored, allowing for a variety of different rate-heterogeneity across sites (RHAS): a proportion of Invariant sites (+I), a discrete Gamma distribution with four rate categories (+G), a FreeRate model (+R), equal/homogenous rates (+E), and combined rates (I + G, I + R). The RHAS models are homotachous (i.e., every site is assumed to evolve at a fixed rate of change irrespective of which edge in the tree the sequence is evolving along (different sites may evolve at different rates of change)), a necessary requirement due to the presence of short exon alignments. For each model of SE investigated, we conducted a full tree search (using the -mtree option), rather than a default fixed starting tree, to prevent entrapment in local optima. All models of substitution were explored for each alignment of amino acids using the full set of RHAS models in addition to unlinked rates (*R, I*R). Optimal frequencies were inferred by comparing empirical frequencies (+F), maximum likelihood (ML)-optimized frequencies (+FO), and frequencies of the substitution model (+FU). The best-fit model of SE for each alignment was chosen based on the Bayesian Information Criterion (BIC) and used to infer an ML gene tree.

#### 2.2.3. Species Tree Inference Using Concatenation and Coalescence Methods

Alignments of both DNA and amino acids were concatenated into a supermatrix using FASconcat [51]. These concatenated datasets (length 1,271,235 bp and 423,745 amino acids) were used to infer the mammalian species tree using the ML method implemented in IQTREE2, partitioned by the best-fit model of SE per-gene, with 1000 bootstrap replicates generated using the ultrafast bootstrap approximation (UFBoot, [52]). The species tree under a coalescent model was inferred using individual gene trees (available at Figshare, https://figshare.com/s/b0bca1ca0f8328ec993a (accessed on 28 March 2022)) with ASTRAL [53] and all concatenated datasets with SVDquartets ([54], 500 bootstrap replicates). Polytomies that were arbitrarily resolved when using IQTREE2 to infer gene trees may impact quartet resolution. Therefore, we explored coalescent trees both with and without polytomies resolved. For all inferred gene trees, any edges with a length less than 0.01 substitutions per site were collapsed into polytomies using the ‘*di2multi*’ function in the R package *ape* [55]. Given the short length of some exons, all analyses were repeated using only alignments greater than 500 bp (n = 157 alignments). To test the fit of all gene trees to all species trees inferred, both Robinson–Foulds distances and tree topology tests between gene tree/species tree combinations were carried out using the *Phangorn* package in R [56] and topology tests in IQTREE2. Where gene trees contained fewer than 47 species, gene trees were compared to a pruned version of the species tree.

## 3. Results

### 3.1. Phylogenetic Inference Using Retrotransposon Presence/Absence Data

**Figure 2:**
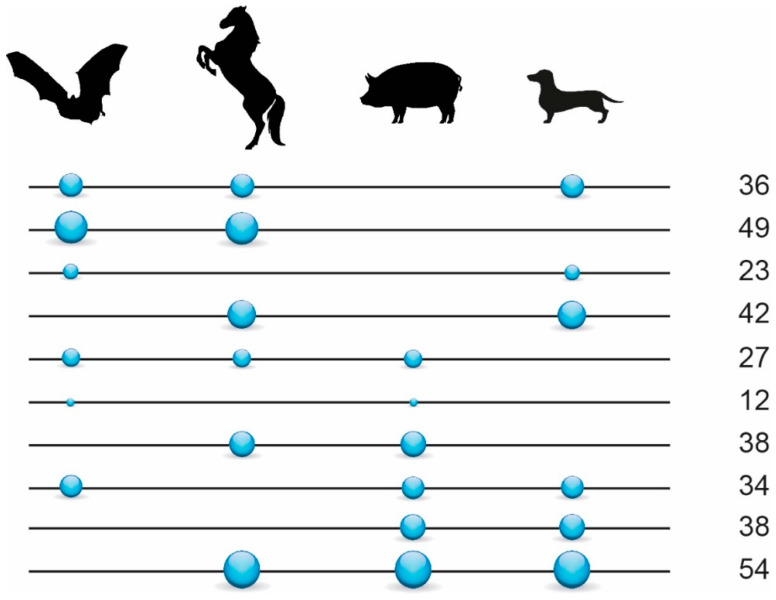
Retrotransposon presence/absence markers in Scrotifera. Values on the right represent the total number of informative retrotransposon insertions found for the respective interordinal affiliations as indicated by the circles (e.g., 36 shared retrotransposons for Chiroptera, Perissodactyla, and Carnivora). Circles on lines represent the presence states of retrotransposons, and the lines without circles represent the absence states. The size of the circles reflects the relative number of diagnostic markers. We used only 353 markers diagnostic for Scrotifera (from 470 in total) for the 4-LIN analysis (represented in the figure).

We investigated the diagnostic presence/absence patterns of LINE1 and LTR retrotransposons extracted from the genome assemblies of the star-nosed mole (148,335 retrotransposon insertions), greater mouse-eared bat (190,326 insertions), horse (325,538 insertions), pig (338,342 insertions), and dog (299,793 insertions). N-way analyses revealed 1910 perfect diagnostic presence/absence patterns. Manual curation of the extracted alignments of their presence/absence loci yielded 367 phylogenetically informative markers (Supplementary Tables S1 and S2, Supplementary File S1). By adding non-overlapping markers found in Doronina et al. [36] to the present dataset, we obtained 470 markers for laurasiatherian relationships (Figure 2, Supplementary Table S1).

**Figure 3:**
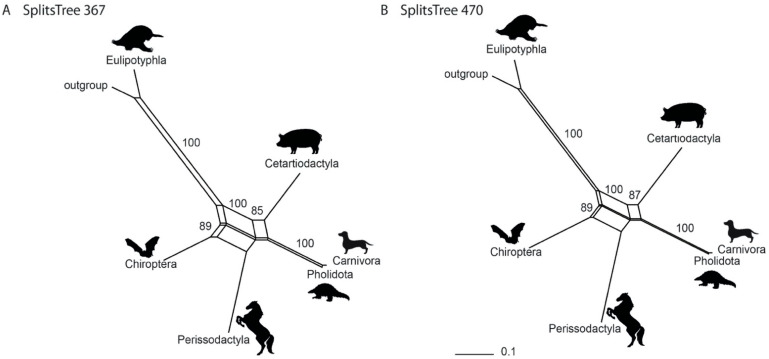
Phylogenetic networks from neighbor-net analyses (SplitsTree4). The datasets of (**A**) 367 and (**B**) 470 retrotransposon markers were analysed. Numbers represent bootstrap values. Branch lengths are indicated below the trees.

The neighbor-net analysis of the 367-marker dataset and the 470-marker dataset identified a phylogenetic network rather than a simple, bifurcating tree (Figure 3). This result is comparable to that of Doronina et al. [36] (also see Supplementary Figure S2), with Chiroptera at the second basal split after Eulipotyphla, a sister-group relationship between Cetartiodactyla and Ferae, and an association of Perissodactyla with both Chiroptera and Cetartioferae (Cetartiodactyla + Ferae).

**Figure 4:**
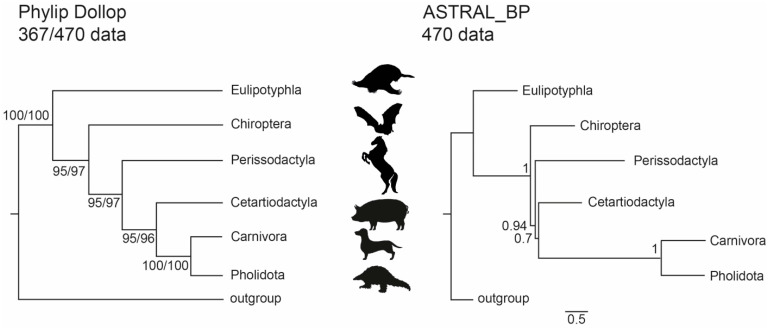
Species tree for Laurasiatheria based on retrotransposon presence/absence data. The tree on the left is the most parsimonious tree with Dollo parsimony (261 and 360 steps for 367- and 470-marker datasets, respectively) and was obtained with the Dollop program in Phylip. The tree on the right was obtained with the coalescence method ASTRAL_BP. Numbers on the left are bootstrap support values; numbers on the right are local posterior probabilities. Dual bootstraps on the left show results for the new 367 TE dataset and the 470 TE dataset after adding non-overlapping data from Doronina et al. [36]. The branch lengths for the ASTRAL_BP tree, in coalescent units, are indicated by the scale bar.

Comparable trees associating Cetartiodactyla and Ferae with Perissodactyla, Chiroptera, and Eulipotyphla as successively more distant relatives of this clade were derived by PHYLIP Dollop (Figure 4) and MrBayes (see Supplementary Figure S2). Interestingly, the tree topology identified by the ILS-aware MSC methods was identical to those inferred by PHYLIP and MrBayes, although mostly with relatively low bootstrap and local posterior probability values (Supplementary Figure S2). However, the results inferred using ASTRID_BP supported a Cetartiodactyla-Ferae affiliation, with bootstrap-support scores above 80%. The local posterior probability support of ASTRAL_BP reconstructions for conflicting affiliations were higher compared to the previously published dataset: i.e., for the 367-dataset, 0.67 for Cetartiodactyla + Ferae, and 0.96 for Perissodactyla + Cetartiodactyla + Ferae; for the 470-dataset, 0.7 for Cetartiodactyla + Ferae and 0.94 for Perissodactyla + Cetartiodactyla + Ferae (Figure 4); for the 102-dataset [36], 0.58 for Cetartiodactyla + Ferae, and 0.66 for Perissodactyla + Cetartiodactyla + Ferae [34]. Both optimal species trees based on SDPquartets supported Fereuungulata but not Cetartioferae. Instead, the SDPquartets species trees recovered Zoomata (367 markers) or a polytomy between Cetartiodactyla, Perissodactyla, and Ferae (470 markers). Bootstrap analyses with SDPquartets recovered Fereuungulata and Cetartioferae with both datasets (Supplementary Figure S2), but support for Cetartioferae was <50% in both cases (Supplementary Figure S2).

Furthermore, the 4-LIN statistical likelihood test revealed an ancestral hybridization/introgression in laurasiatherian history. The Perissodactyla lineage resulted from the fusion of ancestral Chiroptera and Cetartioferae (*p* < 4.7 × 10^−7^ and *p* < 4.4 × 10^−6^ for the datasets with 367 and 470 markers, respectively; Supplementary Figure S2). However, the Quartet-Asymmetry test did not support this and found no significant hybridization/introgression among Carnivora, Cetartiodactyla, Perissodactyla, and Chiroptera (Supplementary Table S3).

### 3.2. Phylogenetic Inference Using Exon Data

**Figure 5:**
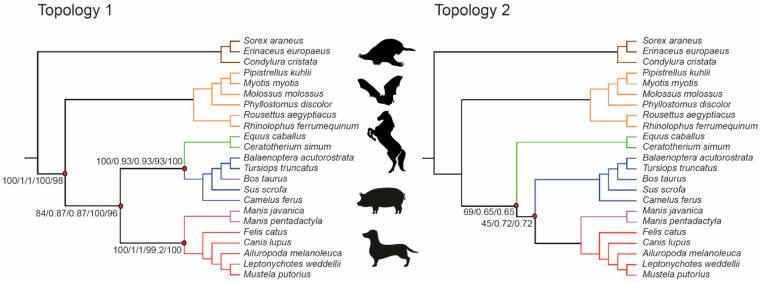
Two laurasiatherian topologies with the highest support revealed by exon data analyses. Support scores for internal edges are displayed only for those edges where at least one dataset provides support < 100% (Topology 1: DNA concatenated ML, DNA coalescence polytomies not collapsed ASTRAL, DNA concatenated SVDquartets, amino acids concatenated ML; Topology 2: DNA (min 500 bp length) concatenated ML, DNA (min 500 bp length) coalescence polytomies not collapsed ASTRAL).

We applied concatenation and coalescence methods to the 9266 exon alignments for DNA and amino acids. The concatenated data with all exons had 327,783 and 60,794 parsimony-informative sites for DNA and amino acids, respectively. Concatenated datasets were partitioned using best-fit models of SE (Supplementary Table S4). A total of 157 genes had alignments greater than 500 bp in length and were used as a separate dataset for both methods (34,216 and 5328 parsimony-informative sites for DNA and amino acids, respectively). Overall, 14 different combinations of data type and phylogenetic method (four using a concatenated dataset, ten using coalescence models, see Table 1) were used to infer species trees, all of which were rooted between Atlantogenata and Boreoeutheria. Four of the analyses employed maximum likelihood to infer a species tree from a concatenated matrix, and ten analyses employed a coalescence method (ASTRAL or SVDquartets) to infer a species tree. Seven distinct laurasiatherian topologies were identified across all 14 trees (Figure 5, Supplementary Figure S3): Eulipotyphla sister to Scrotifera, Chiroptera sister to Fereuungulata, and Euungulata sister to Ferae (Topology 1, represented by five datasets); Eulipotyphla sister to Scrotifera, Chiroptera sister to Fereuungulata, Perissodactyla sister to Cetartioferae, Cetartiodactyla sister to Ferae (Topology 2, represented by two datasets); Eulipotyphla sister to Scrotifera, with Chiroptera + Cetartiodactyla and Perissodactyla + Ferae as sister clades (Topology 3, represented by one dataset); Eulipotyphla + Chiroptera sister to Fereuungulata, Cetartiodactyla sister to Zoomata, Pholidota sister to Carnivora + Perissodactyla (Topology 4, represented by one datasets); Eulipotyphla + Chiroptera sister to Fereuungulata, Euungulata sister to Ferae (Topology 5, represented by one dataset); Eulipotyphla sister to Scrotifera, Chiroptera sister to Fereuungulata, Cetartiodactyla sister to Perissodactyla + Ferae (Topology 6, represented by three datasets) and Eulipotyphla sister to Scrotifera, Chiroptera sister to Fereuungulata with Cetartiodactyla + Pholidota sister to Perissodactyla + Carnivora (Topology 7, represented by one dataset). We note that Topology 1 is identical to the Laurasiatheria topology inferred by Jebb et al. [25] in most of their analyses; this topology also had the highest support values for laurasiatherian orders relative to other topologies. Topologies 1, 2, and 3 remained unchanged relative to Jebb et al. [25] for relationships within Atlantogenata and Euarchontoglires. Topology 4 and 6 displayed intraordinal differences within Primates and Rodentia relative to Jebb et al. [25]. In Topology 5, Lagomorpha (sister to Primates + Rodentia) and Afrosoricida (sister to all other afrotherian orders) were in different positions than in Jebb et al. [25], while interordinal differences within Rodentia were observed in Topology 7.

When comparing Robinson–Foulds (RF) distances between gene trees and each of the five species topologies, median distances of 46 and 58 were found for DNA and amino acid gene trees irrespective of the target, possibly due to the removal of key taxa differentiating different species tree topologies and rendering RF distances largely uninformative for our analyses. A total of 9105 DNA and 6040 amino acid ‘gene trees’ favoured one topology over the other four (Table S1, Supplementary Figure S4), with more DNA alignments supporting Topology 5 and more amino acid alignments supporting Topology 4. Topology 5 maintains Fereuungulata but represents Chiroptera as a sister clade to Eulipotyphla, albeit with low bootstrap support (65.8), while Topology 4 has some of the lowest support overall for laurasiatherian nodal splits.

## 4. Discussion

Consistent with a previous but less extensive phylogenetic study of retrotransposons [36], we found presence/absence markers supporting all possible interordinal affiliations within Scrotifera (Figure 2, Supplementary Tables S1 and S2). Compared to Doronina et al. [36], the new screening benefits from the higher genome quality of chiropteran taxa, revealing twice as many markers. However, our current screening did not retrieve 67 of the previous markers from Doronina et al. [36] that have a clear presence/absence state in Eulipotyphla, despite similar screening stringency and improved bat assemblies. One explanation for these differences is that the basic two-way genome alignments used different representatives for Chiroptera (greater mouse-eared bat instead of little brown bat) and Cetartiodactyla (pig instead of cow). For example, locus D_C0047 from Doronina et al. [36] is missing in the pig genome, and locus DB_0042 is omitted from the derived two-way genome alignments. Furthermore, the new 2-n-way screening protocol considered only loci with unmistakable presence or absence states in the star-nosed mole, whereas Doronina et al. [36] did not include Eulipotyphla in the computational screening but subsequently added an available representative of Eulipotyphla via Blast. This shows that assemblies of different qualities can influence both character and character state inferences. However, the diagnostic signals of retrotransposon markers found in both studies lead individually or in combination to the same phylogenetic tree and represent the up-to-date largest retrotransposon phylogenetic datasets for laurasiatherians.

Within Scrotifera, the new 367 and combined 470 retrotransposon markers (including markers from Doronina et al. [36]) recovered strong support for a basal split between Chiroptera and Fereuungulata. Most analyses also showed Perissodactyla as the sister taxon to a monophyletic group comprising Ferae + Cetartiodactyla (Figure 4, Supplementary Figure S2). A 4-LIN significance test [38] further suggests that ancestral hybridization/introgression between Chiroptera and Cetartioferae created or at least contributed to the modern clade Perissodactyla. However, a Quartet-Asymmetry test [34] failed to confirm this hybridization/introgression scenario. It should be noted that the Quartet-Asymmetry test used a significantly reduced dataset (202 markers vs. 353 markers) because of methodical pairwise presence or absence characters sorted in quartets. On the contrary, the 4-LIN test takes into account not only pairwise order affiliations but also triplets (e.g., markers with [+ + + –] states).

When using coding regions to infer the tree topology of Laurasiatheria, single-exon alignments are preferable to complete protein-coding sequences because they are more likely to satisfy the coalescence gene assumption, i.e., there is recombination between c-genes but not within c-genes [34]. Applying high-quality exon screens to the mammalian assemblies resulted in 9266 exon alignments that were processed with coalescence and concatenated methods. In an extensive search for the best-fit models of SE, we identified five laurasiatherian topologies with varying levels of support (coalescent units, bootstrap values). Topology 1, with Eulipotyphla sister to Scrotifera, followed by Chiroptera sister to Fereuungulata (Figure 5), was the species tree recovered using the complete concatenated datasets for DNA and amino acid sequences with the maximum likelihood method. Using all 9266 gene trees with the coalescence method implemented in ASTRAL recovered two different topologies based on DNA (Topology 1) and amino acid (Topology 2) sequences. Topology 2 represents Eulipotyphla sister to Scrotifera, followed by Chiroptera diverging from Fereuungulata; Perissodactyla are the sister group of Cetartioferae (Figure 5).

One likely source for the disparity of DNA and amino acids is that there are fewer parsimony-informative sites in our amino acid alignments (median: four sites) compared to DNA (median: 28 sites). The longer an alignment of variable sites is, the more likely it is that a consistent phylogenetic estimate can be inferred from the data [57]. While the laurasiatherian topology with the highest support values (Topology 1) has been recovered by sequence data previously [25], the fact that alternative topologies with different levels of support exist implies that some features of mammalian evolution are not being modelled accurately by current methods. Additionally, stochastic error may interfere with the inference of optimal topologies. When using only exons greater than 500 bp, Topology 1 was never recovered, despite fitting the size requirement expected for a c-gene, suggesting that 157 exons may not be a large enough dataset to resolve Laurasiatheria. Finally, we note that the RHAS models used here are homotachous. If the evolutionary processes were heterotachous, we should expect model misspecification in the current estimates. In this case, heterotachous models could be considered, but they require a larger number of sites and are therefore not applicable to the analysis of exon alignments as c-genes.

Identifying the interordinal relationships within Laurasiatheria is an enduring challenge. ASTRAL_BP analysis of the retrotransposon datasets suggests that the four-lineage polytomy is resolved as a pectinate tree (Topology 2) with perissodactyls and chiropterans as successively more distant outgroups to Cetartioferae. Indeed, consecutive short branch lengths on the ASTRAL_BP species trees for both the 367-marker dataset (x = 0.149, y = 0.0615) and the 470-marker dataset (x = 0.1151, y = 0.0551) are both in the anomaly zone [1]. If the retrotransposon tree is correct, then we might expect concatenation to fail and recover a symmetric tree for the four major lineages [1]. This is the case for the concatenated analysis with amino acid data and 157 larger exons that supports Topology 3 (Chiroptera + Cetartiodactyla sister to Zoomata), but other maximum likelihood analyses with concatenated datasets support pectinate resolutions of the polytomy that are in agreement with analyses of the same datasets with the summary coalescence method ASTRAL, i.e., nucleotide datasets with 9266 and 157 exons. There is also an example of different coalescence methods (ASTRAL, SVDquartets) recovering different topologies with the same data, i.e., a nucleotide dataset with 157 larger exons. Therefore, the contrasting results of the various analyses are more complex than concatenation versus coalescence and the effects of the anomaly zone.

The independent whole-genome analyses of retrotransposon presence/absence patterns and exon/amino acid sequences consistently revealed Chiroptera as the second split after basal Eulipotyphla. This result agrees with many previous large-scale sequence studies (e.g., [24,25,58]). However, both data types contain signals supporting incongruent tree topologies for the remaining scrotiferans. This is probably due to extensive ILS and possibly hybridization/introgression. The current study revealed two dominant tree topologies for retrotransposon data: (Perissodactyla (Cetartiodactyla, Ferae)) [Cetartioferae hypothesis] and the exon sequence data: (Ferae (Cetartiodactyla, Perissodactyla)) [Euungulata hypothesis]. The sequence-based Euungulata clade was also previously supported by other sequence data (e.g., [18]). The retrotransposon-based Cetartioferae clade was indicated in the earlier presence/absence study of Doronina et al. [36]. Cetartioferae also received support from an intron-based analysis of Chen et al. [17]. Interestingly, reducing the herein applied exon dataset to the 157 largest exon sequences also retrieved support for Cetartioferae. Further analyses might shed more light on which of these two topologies better describes the early diversification of Scrotifera.

## 5. Conclusions

Our in-depth analyses of novel high-quality retrotransposon and exon data have revealed that biological processes such as rapid speciation and ILS may underpin phylogenetic conflict within Laurasiatheria. Although past analyses had already explored ILS and model misspecification as drivers of phylogenetic incongruence, the current analysis extends these considerations by using novel high-quality models of retrotransposons and exons, as well as extensive, in-depth analyses of these data. Unfortunately, our discordant results do not lay the laurasiatherian problem to rest, suggesting that adequate modelling of rapid branching events remains a challenge in phylogenomics. These “hard-nodes” still remain problematic, but attempting to resolve them using different data types (e.g., indels, intron sequences, viral inserts, and nuclear copies of mitochondrial DNA (numts)) and ILS- and hybridization-aware methods will stimulate the development of novel strategies and datasets that may eventually recover the true evolutionary history of life.

## Figures and Tables

**Table 1:** Exon datasets and methods used to construct the laurasiatherian topology. Datasets both with (9266 exons) and without a minimum length of 500 bp (157 exons) are displayed. The interordinal clades within Laurasiatheria with the lowest bootstrap/posterior probability values are also displayed. NT denotes nucleotide, AA denotes amino acid, SM indicates supermatrix, p.c. indicates branches < 0.01 substitutions per site collapsed into polytomies, b.n.c.—branches not collapsed, ML —maximum likelihood, Car—Carnivora, Pho—Pholidota, Fer—Ferae, Cet—Cetartiodactyla, Per—Perissodactyla, Chi—Chiroptera, Eul—Eulipotyphla.

Seq Type	Data Type	Exon Dataset	Method	Topology	Lowest Bootstrap/Local Posterior Probability Values
NT	Concatenated SM	9266	ML	1	84 (Fer(Per,Cet))
NT	Coalescence, b.n.c.	9266	ASTRAL	1	0.87 (Fer(Per,Cet))
NT	Concatenated SM	9266	SVDquartets	1	93 (Per,Cet)
NT	Concatenated SM	157	ML	2	45 (Fer,Cet)
NT	Coalescence, b.n.c.	157	ASTRAL	2	0.65 (Per(Fer,Cet))
NT	Concatenated SM	157	SVDquartets	5	65.8 (Chi,Eul)
AA	Concatenated SM	9266	ML	1	96 (Per,Cet)
AA	Coalescence, b.n.c.	9266	ASTRAL	6	0.5 (Car,Pho)
AA	Concatenated SM	157	ML	3	46 (Cet,Chi)
AA	Coalescence, b.n.c.	157	ASTRAL	4	0.35 (Cet(Pho(Per,Car)))
NT	Coalescence, p.c.	9266	ASTRAL	1	0.59 (Fer(Per,Cet))
AA	Coalescence, p.c.	9266	ASTRAL	6	0.59 (Per,Fer)
NT	Coalescence, p.c.	157	ASTRAL	6	0.46 (Cet(Per,Fer))
AA	Coalescence, p.c.	157	ASTRAL	7	0.24 (Cet,Pho)

## Data Availability

All retrotransposon data are presented in Supplementary Materials. The exon DNA and amino acid alignments and individual gene trees are deposited at https://figshare.com/s/b0bca1ca0f8328ec993a (accessed on 28 March 2022).

## Supplementary Materials

The following supporting information can be downloaded at: https://www.mdpi.com/article/10.3390/genes13050766/s1, Figure S1: a flowchart for processing of exon data; Figure S2: phylogenetic tree reconstructions based on retrotransposon datasets; Figure S3: seven different laurasiatherian topologies recovered across 14 datasets using both maximum likelihood- and coalescence-based methods; Figure S4: the number of (A) DNA and (B) amino acid alignments supporting each topology inferred using exon data; Table S1: the number of retrotransposon markers found for all possible laurasiatherian order affiliations; Table S2: presence/absence table of informative retrotransposon markers; Table S3: Quartet-Asymmetry test; Table S4: exon analyses information; File S1: alignments of 367 retrotransposon markers for laurasiatherian relationships; File S2: presence/absence (1/0) data matrices for 367 and 470 retrotransposon datasets.
