# Supplementary material for: Contradictory Phylogenetic Signals in the Laurasiatheria Anomaly Zone
Source: Genes (Basel). 2022 Apr 26;13(5):766. doi: 10.3390/genes13050766 (PMC9141728; doi:10.3390/genes13050766)
Supplement: Supplementary file 1 [file genes-13-00766-s001.zip › Supplementary_Materials/Supplementary_Figure_S4.pdf]

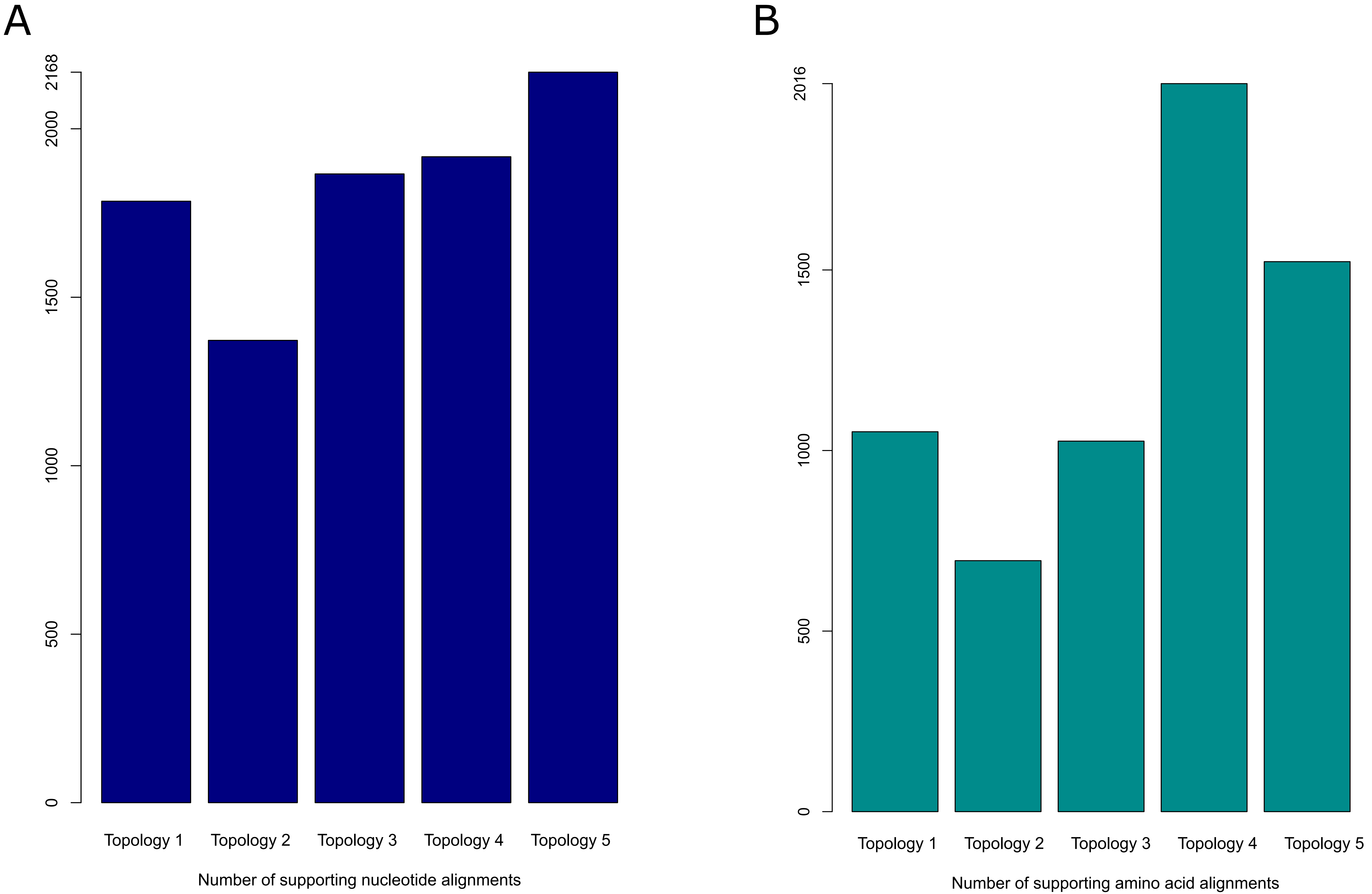

A
2168
2000
1500
1000
500
0
Topology 1
Topology 2
Topology 3
Topology 4
Topology 5
Number of supporting nucleotide alignments
B
2016
1500
1000
500
0
Topology 1
Topology 2
Topology 3
Topology 4
Topology 5
Number of supporting amino acid alignments

Supplementary Figure S4. The number of (A) exon DNA and (B) amino acid alignments supporting each topology inferred using exon data. The best-fit tree topology for each DNA and protein exon alignment was determined using IQTREE2. The total number of alignments supporting each topology is displayed. Only alignments supporting one clear topology over the other were considered (9,105 and 6,040 DNA and amino acid alignments, respectively).
