# Supplementary material for: Contradictory Phylogenetic Signals in the Laurasiatheria Anomaly Zone
Source: Genes (Basel). 2022 Apr 26;13(5):766. doi: 10.3390/genes13050766 (PMC9141728; doi:10.3390/genes13050766)
Supplement: Supplementary file 1 [file genes-13-00766-s001.zip › Supplementary_Materials/Supplementary_Figure_S1.pdf]

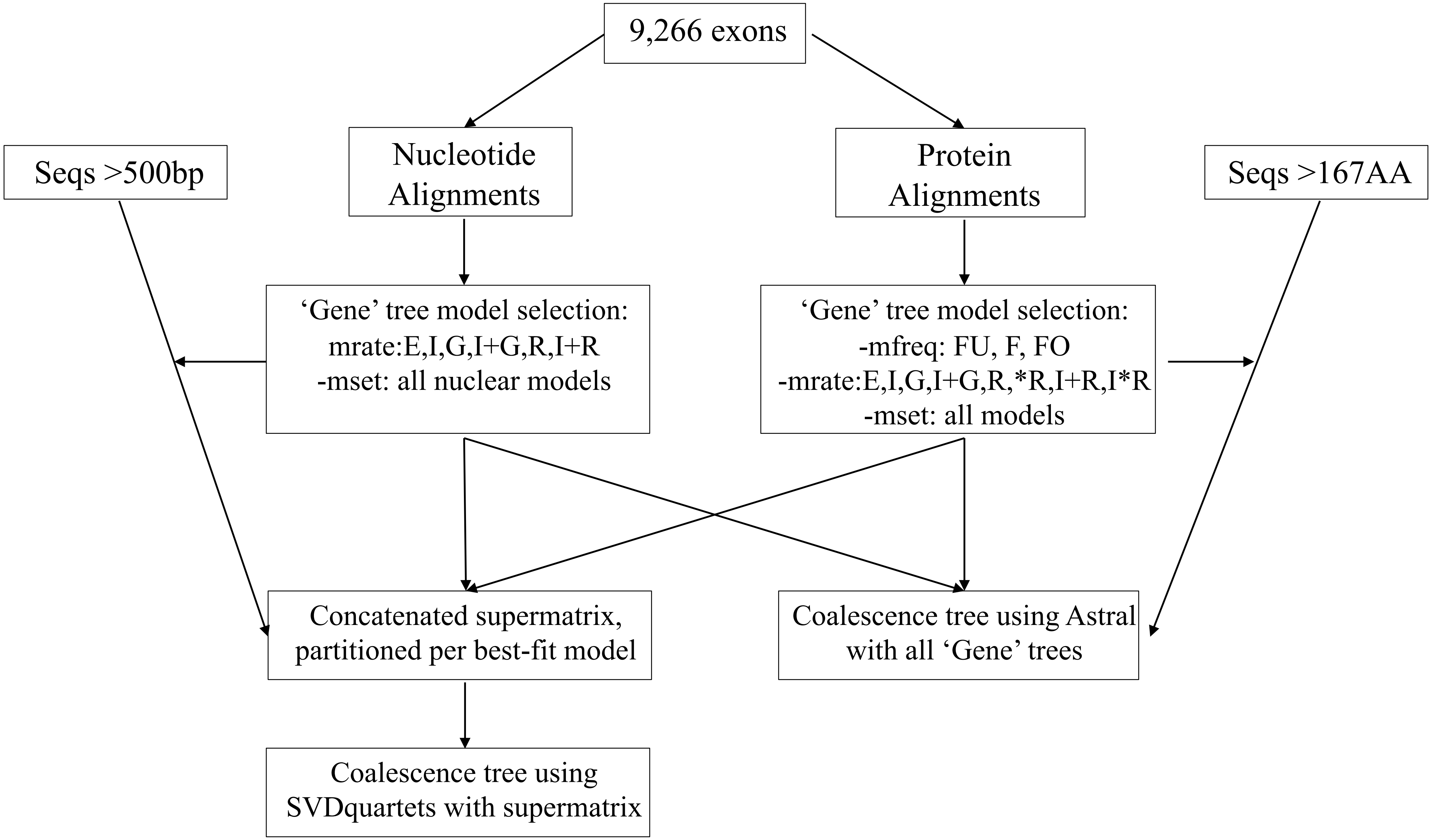

Supplementary Figure S1. A flowchart for processing of exon data. The datasets and the methods applied to them are displayed.
