# Supplementary material for: Contradictory Phylogenetic Signals in the Laurasiatheria Anomaly Zone
Source: Genes (Basel). 2022 Apr 26;13(5):766. doi: 10.3390/genes13050766 (PMC9141728; doi:10.3390/genes13050766)
Supplement: Supplementary file 1 [file genes-13-00766-s001.zip › Supplementary_Materials/Supplementary_Figure_S3.pdf]

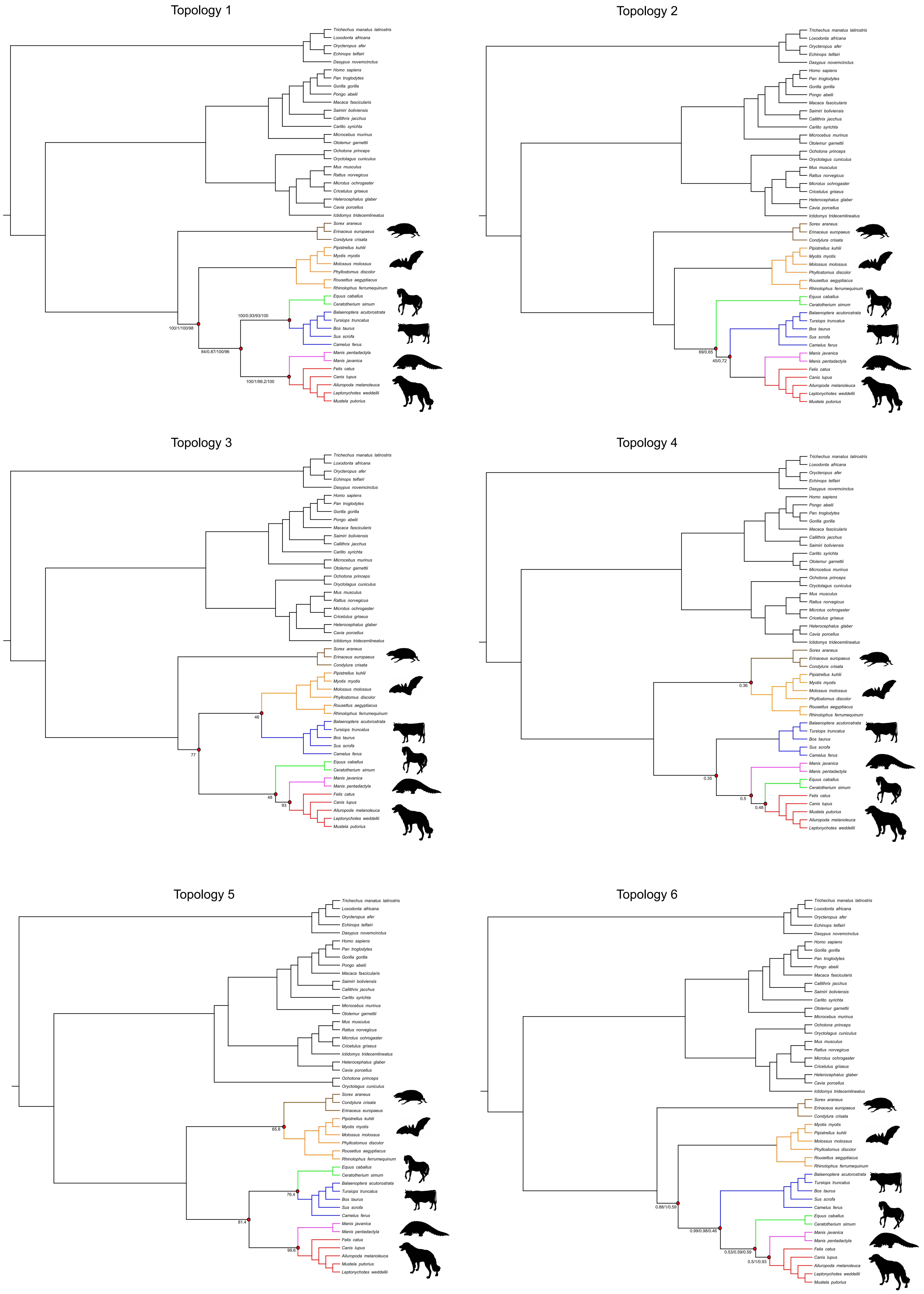

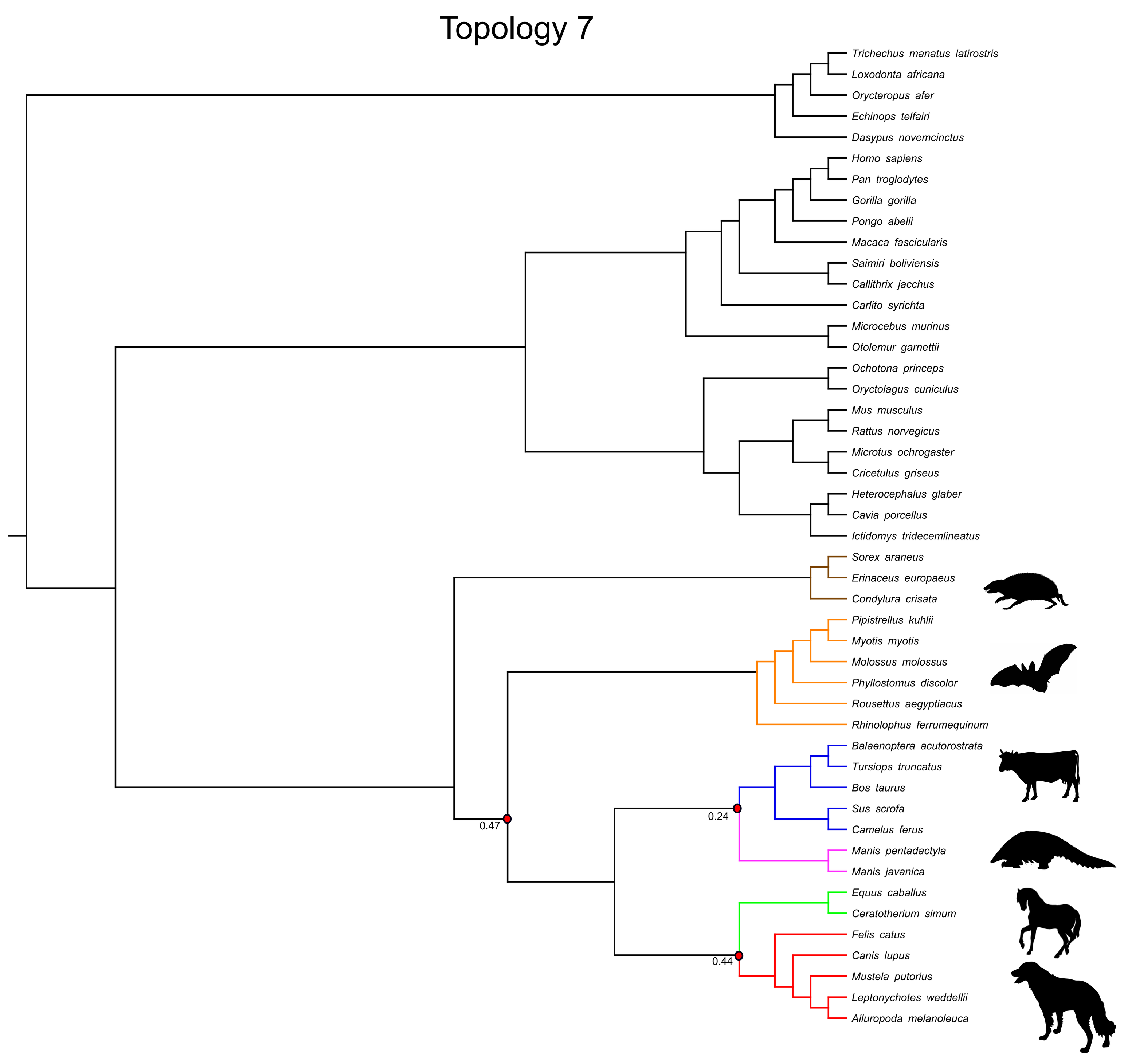

Supplemental Figure S3. Seven different laurasiatherian topologies recovered across 14 datasets using both maximum likelihood and coalescence-based methods. A total of seven distinct laurasiatherian topologies were recovered using both exon DNA and amino acid alignments. Both maximum likelihood and coalescence-based methods were applied. Support values for internal edges where at least one dataset had less than full support are displayed.
