# Supplementary material for: Contradictory Phylogenetic Signals in the Laurasiatheria Anomaly Zone
Source: Genes (Basel). 2022 Apr 26;13(5):766. doi: 10.3390/genes13050766 (PMC9141728; doi:10.3390/genes13050766)

Supplementary Figures S2
Phylogenetic tree reconstructions
based on retrotransposon datasets

A MrBayes 367

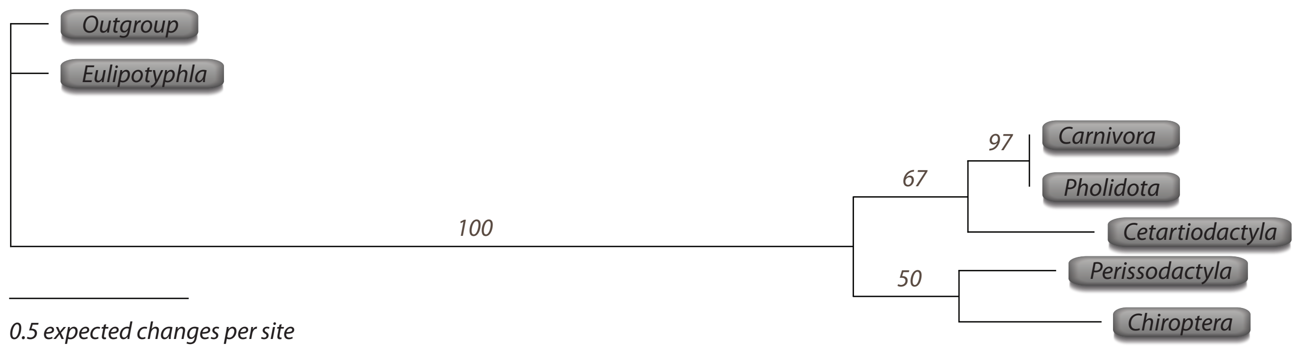

B MrBayers 470

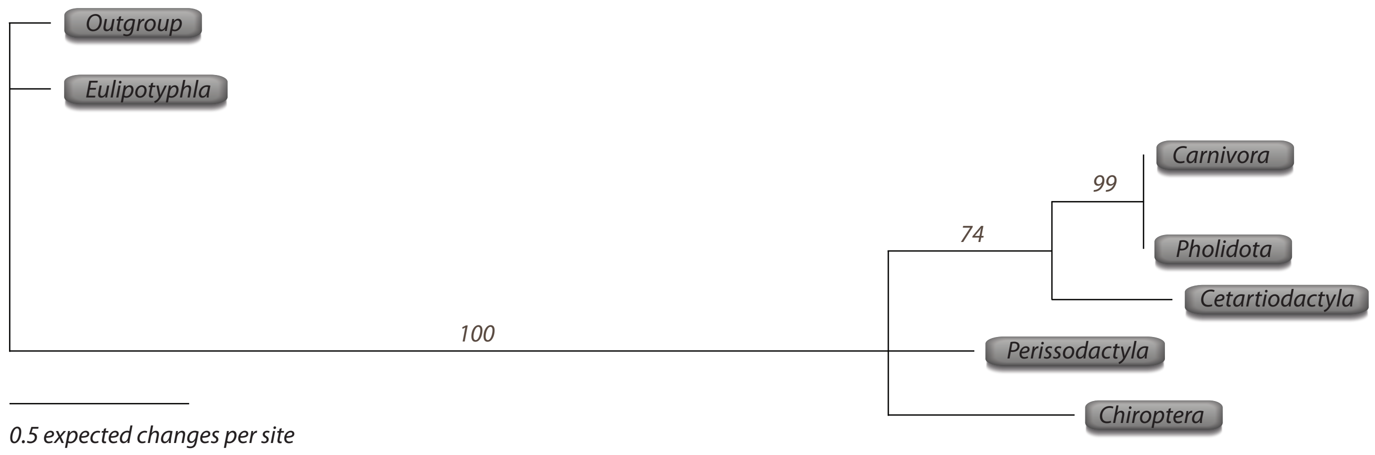

Figure S2a. Bayesian tree reconstruction based on the 367-marker dataset (A) and the 470-marker dataset (B). Numbers represent the Bayesian posterior probabilities (shown as percentages).

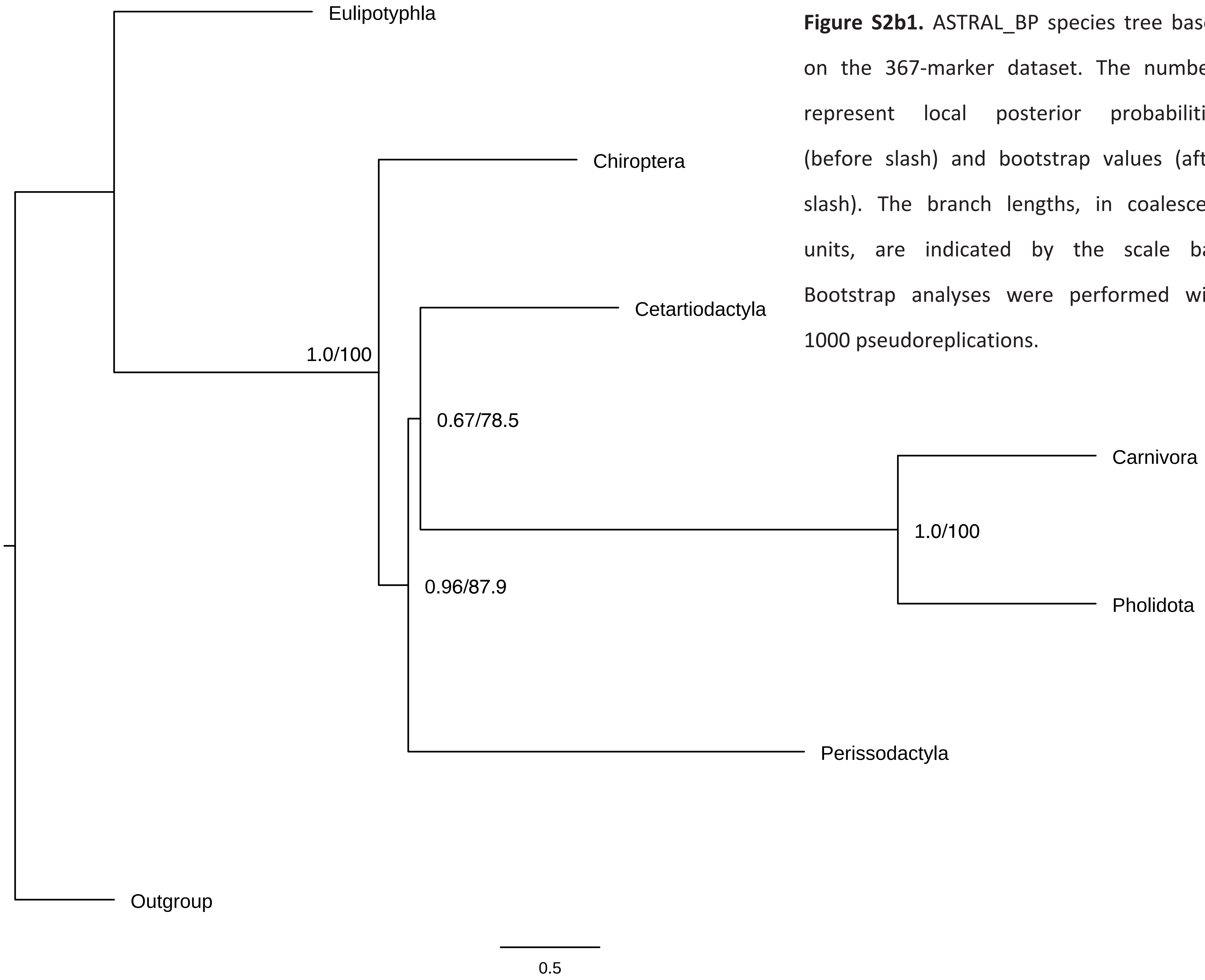

Figure S2b1. ASTRAL_BP species tree based on the 367-marker dataset. The numbers represent local posterior probabilities (before slash) and bootstrap values (after slash). The branch lengths, in coalescent units, are indicated by the scale bar. Bootstrap analyses were performed with 1000 pseudoreplications.

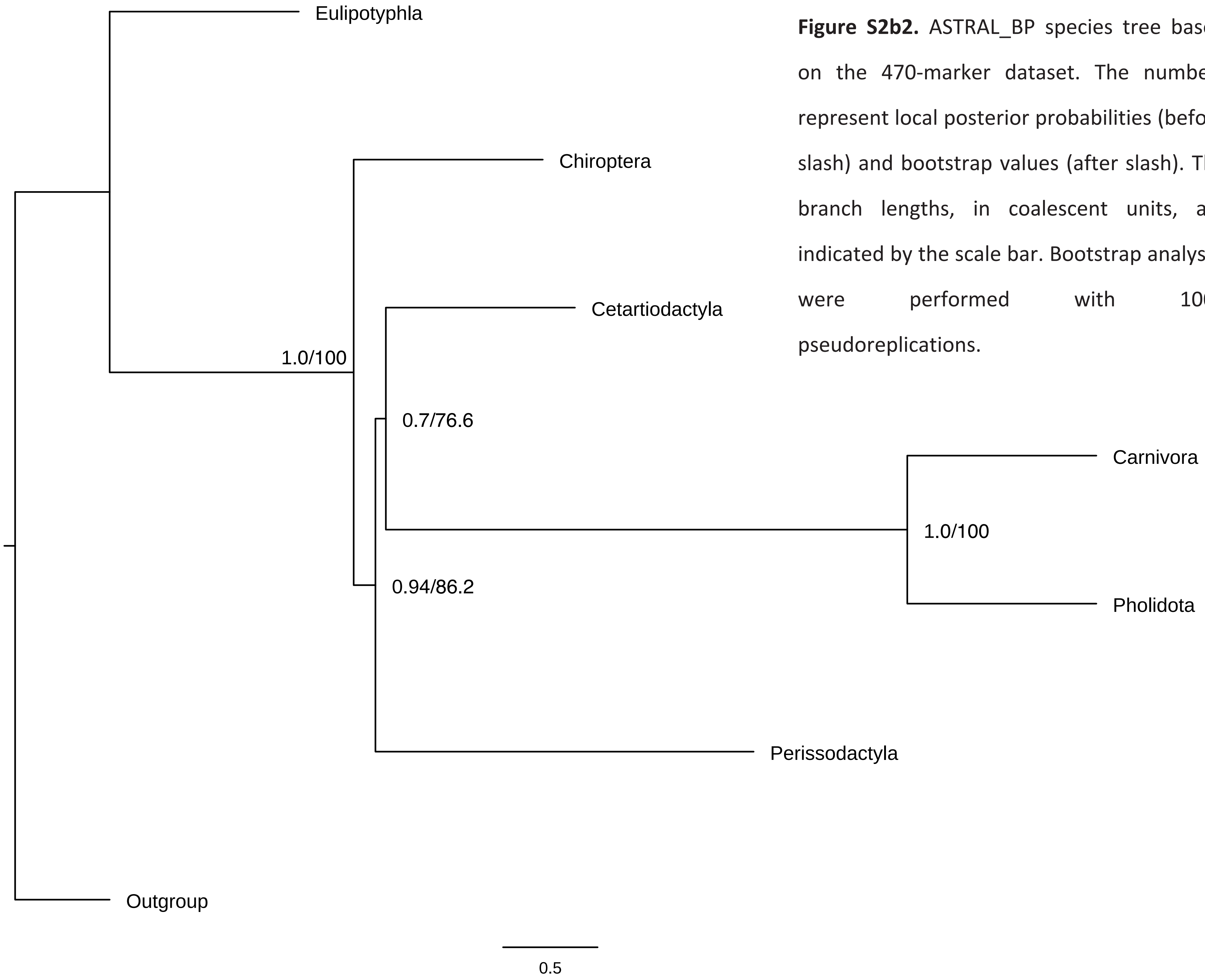

Figure S2b2. ASTRAL_BP species tree based on the 470-marker dataset. The numbers represent local posterior probabilities (before slash) and bootstrap values (after slash). The branch lengths, in coalescent units, are indicated by the scale bar. Bootstrap analyses were performed with 1000 pseudoreplications.

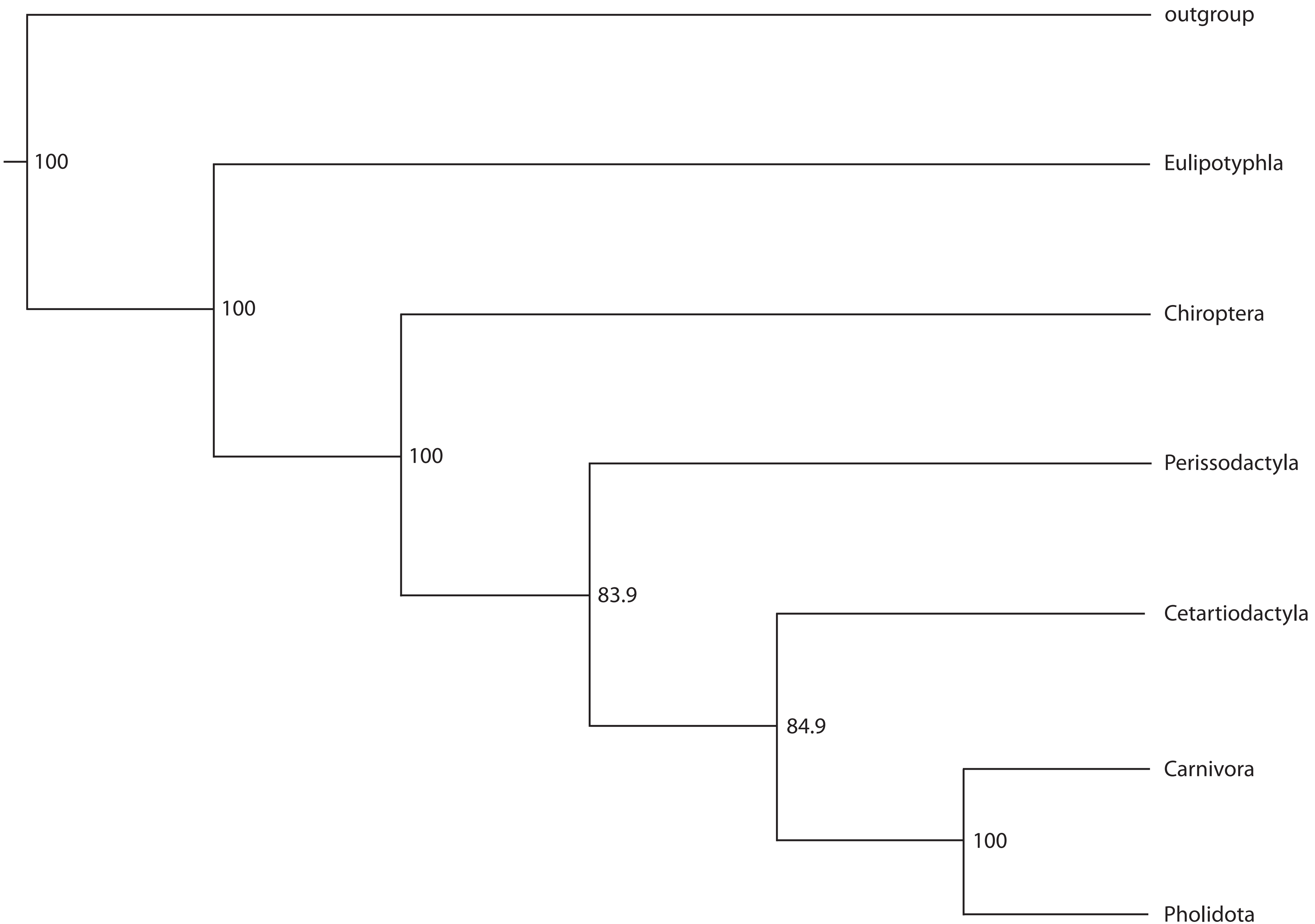

Figure S2c1. ASTRID_BP species tree based on the 367-marker dataset. The numbers indicate bootstrap values. Bootstrap analyses were performed with 1000 pseudoreplications.

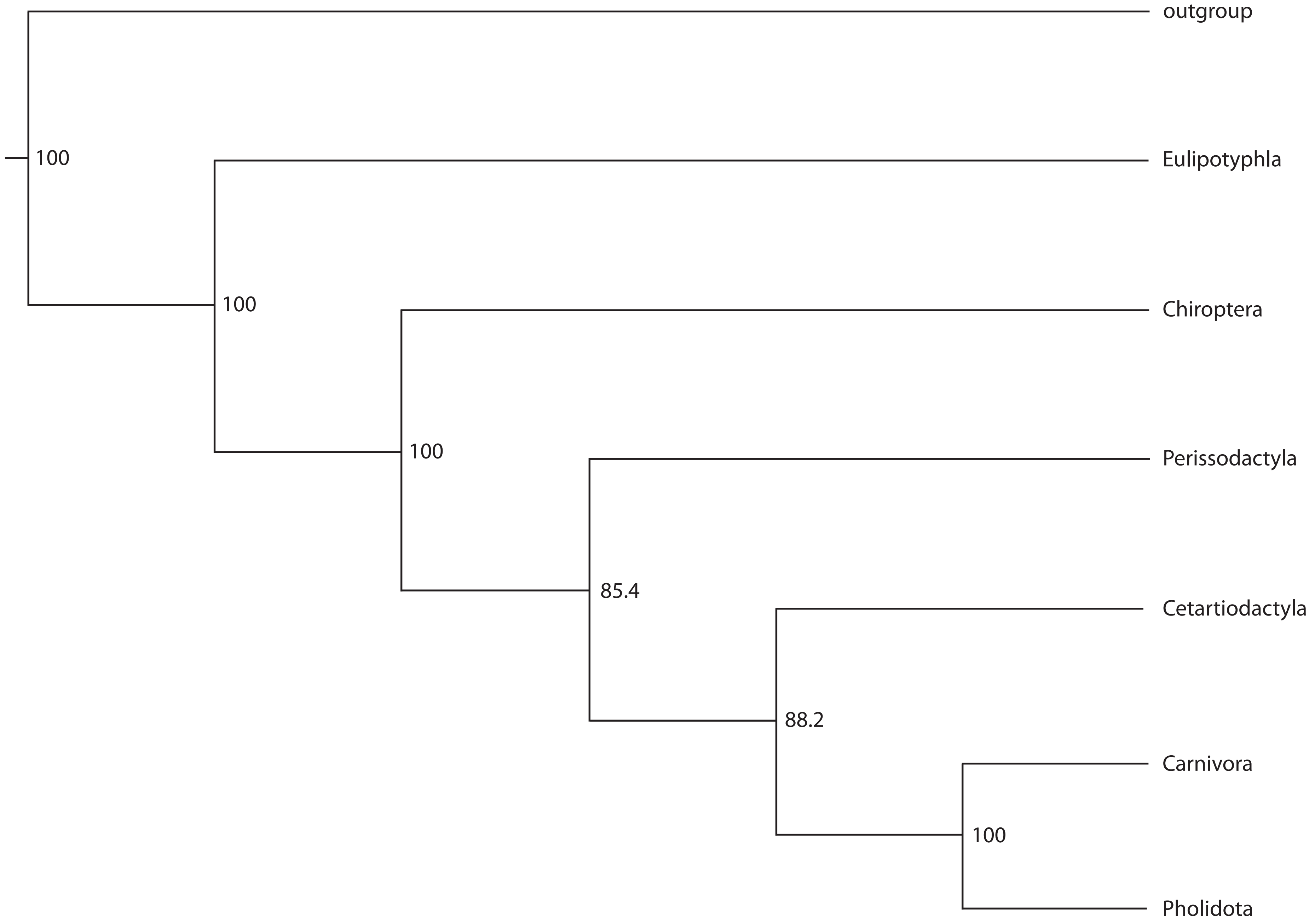

Figure S2c2. ASTRID_BP species tree based on the 470-marker dataset. The numbers indicate bootstrap values. Bootstrap analyses were performed with 1000 pseudoreplications.

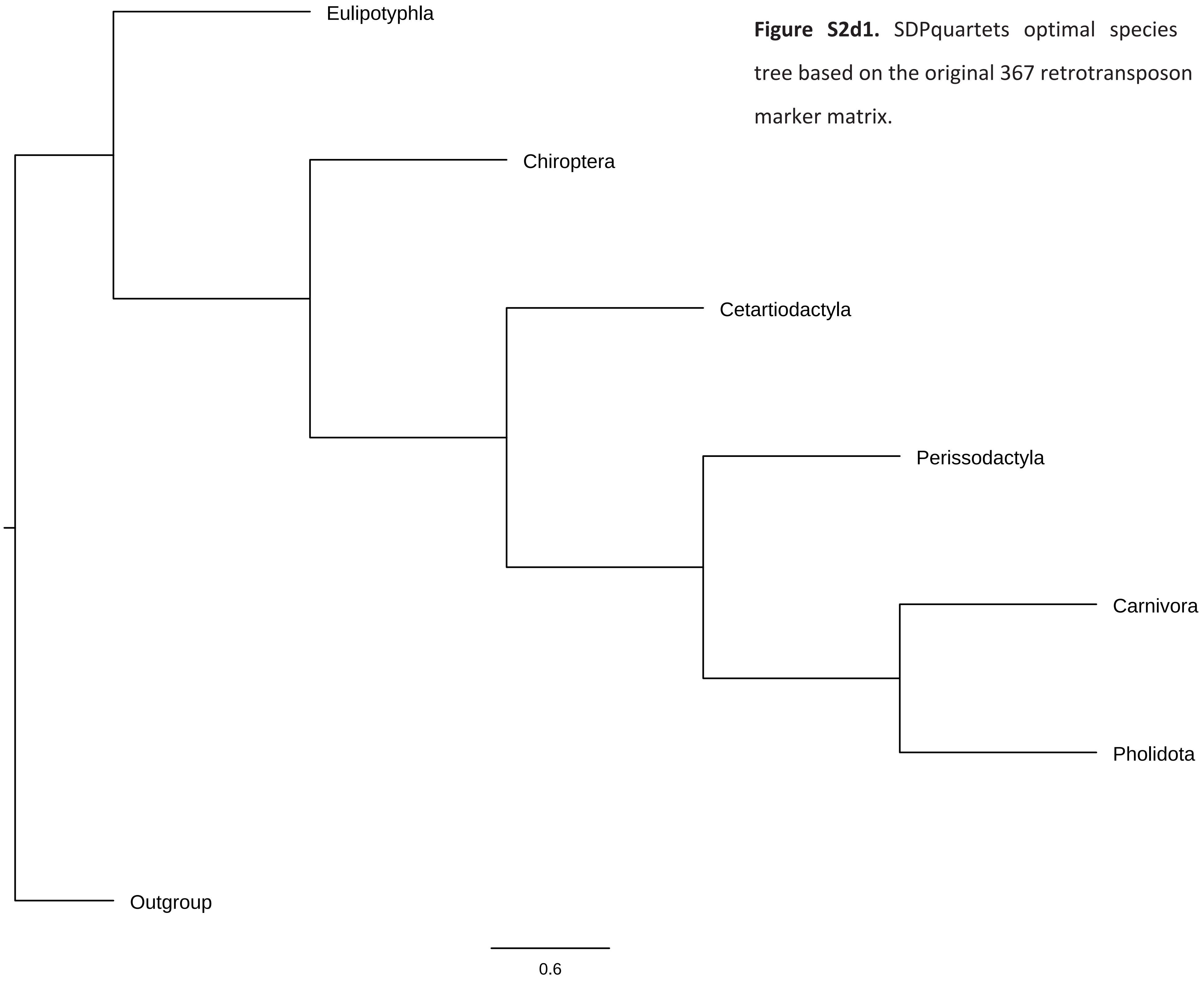

Figure S2d1. SDPquartets optimal species tree based on the original 367 retrotransposon marker matrix.

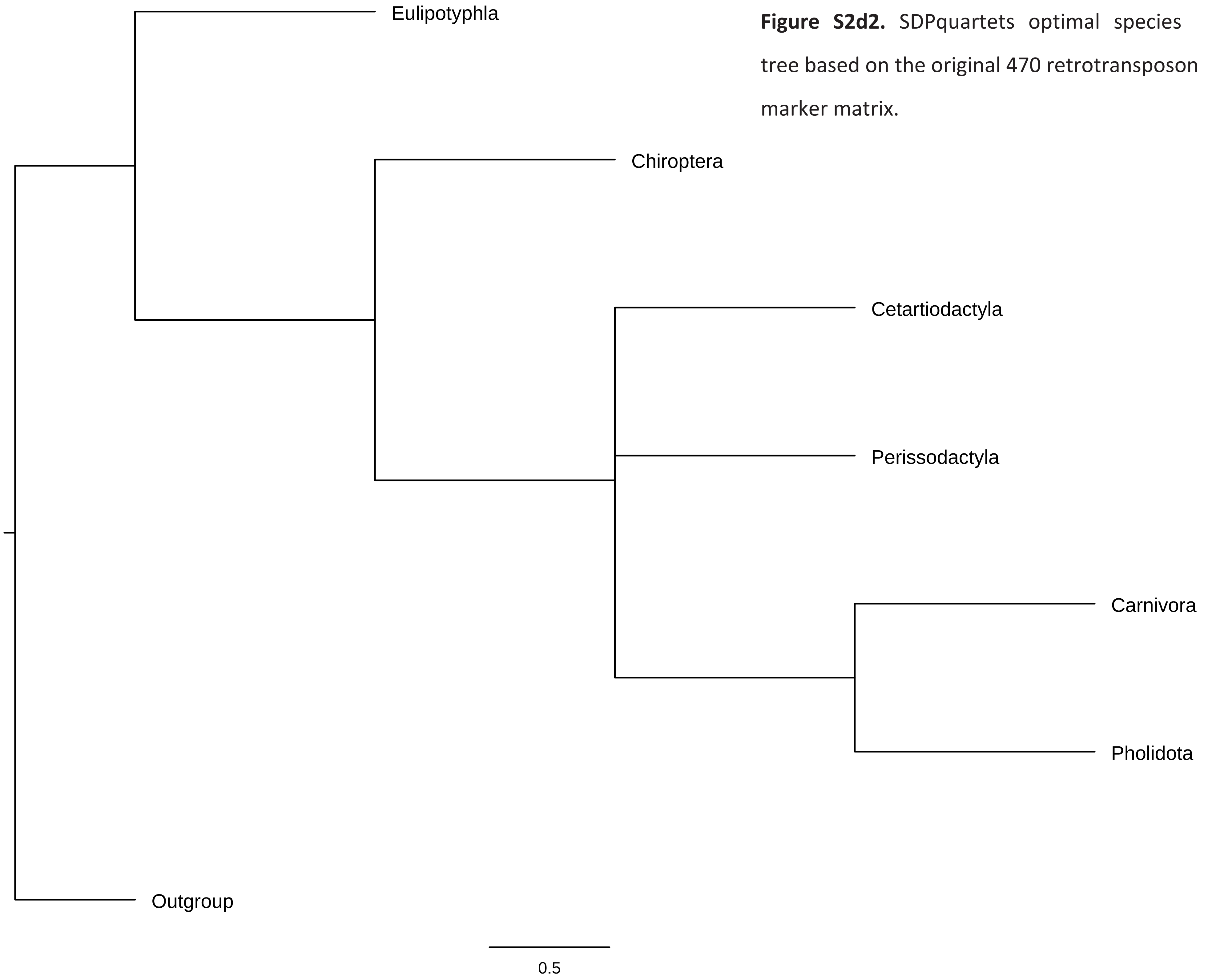

Figure S2d2. SDPquartets optimal species tree based on the original 470 retrotransposon marker matrix.

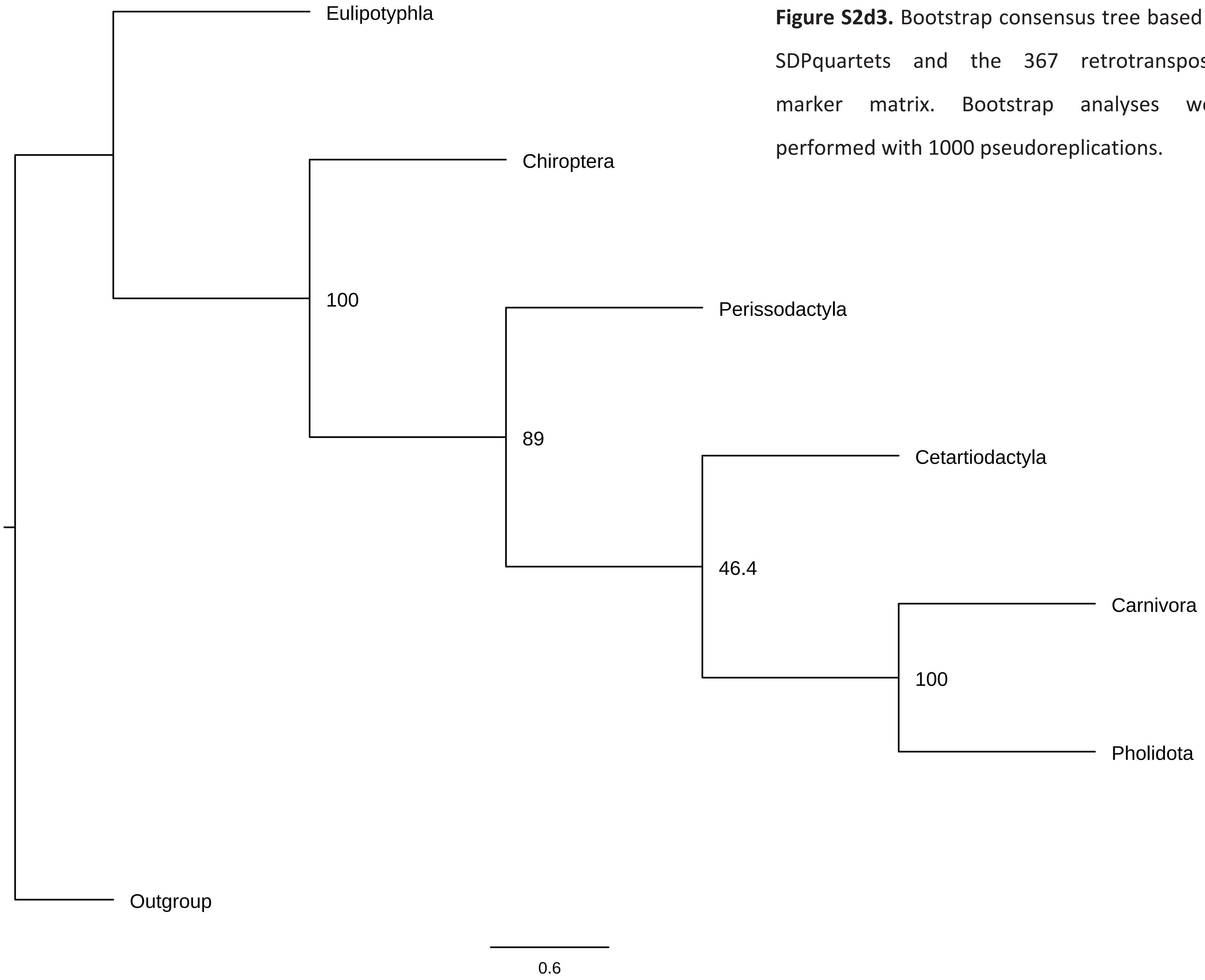

Figure S2d3. Bootstrap consensus tree based on SDPquartets and the 367 retrotransposon marker matrix. Bootstrap analyses were performed with 1000 pseudoreplications.

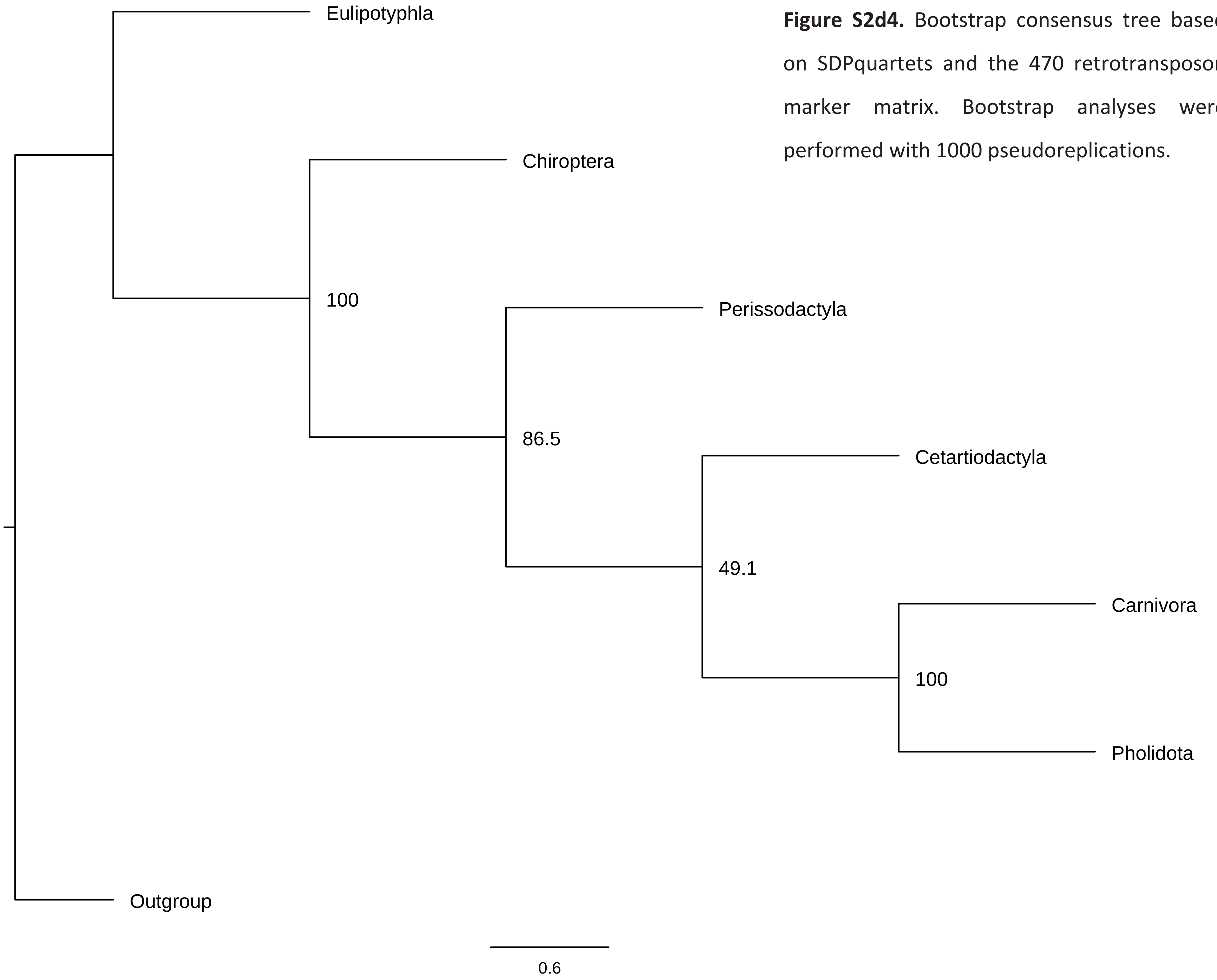

Figure S2d4. Bootstrap consensus tree based on SDPquartets and the 470 retrotransposon marker matrix. Bootstrap analyses were performed with 1000 pseudoreplications.

Figure S2e1. 4-LIN statistical test applied to 367-marker dataset.

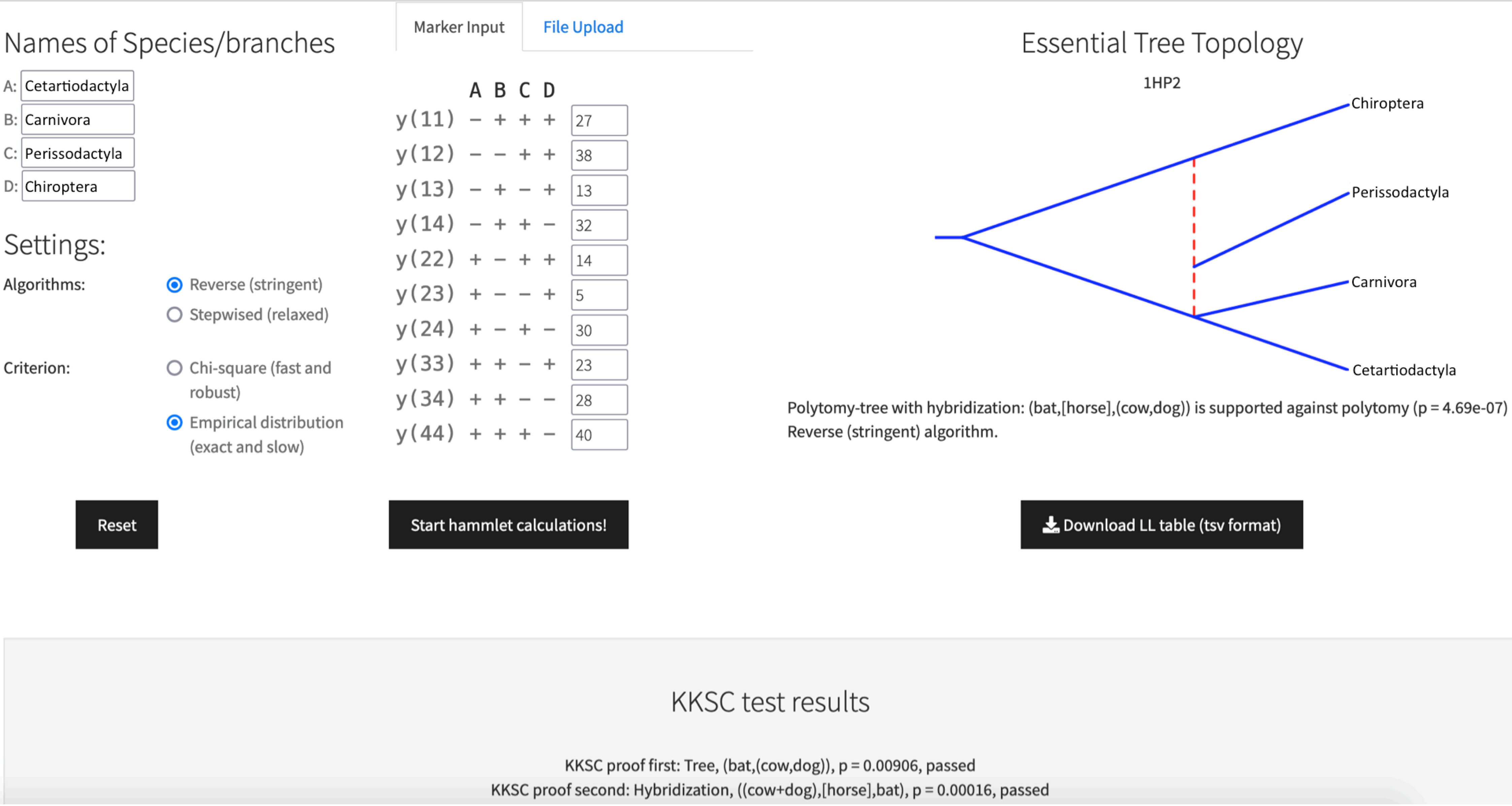

Figure S2e2. 4-LIN statistical test applied to 470-marker dataset.

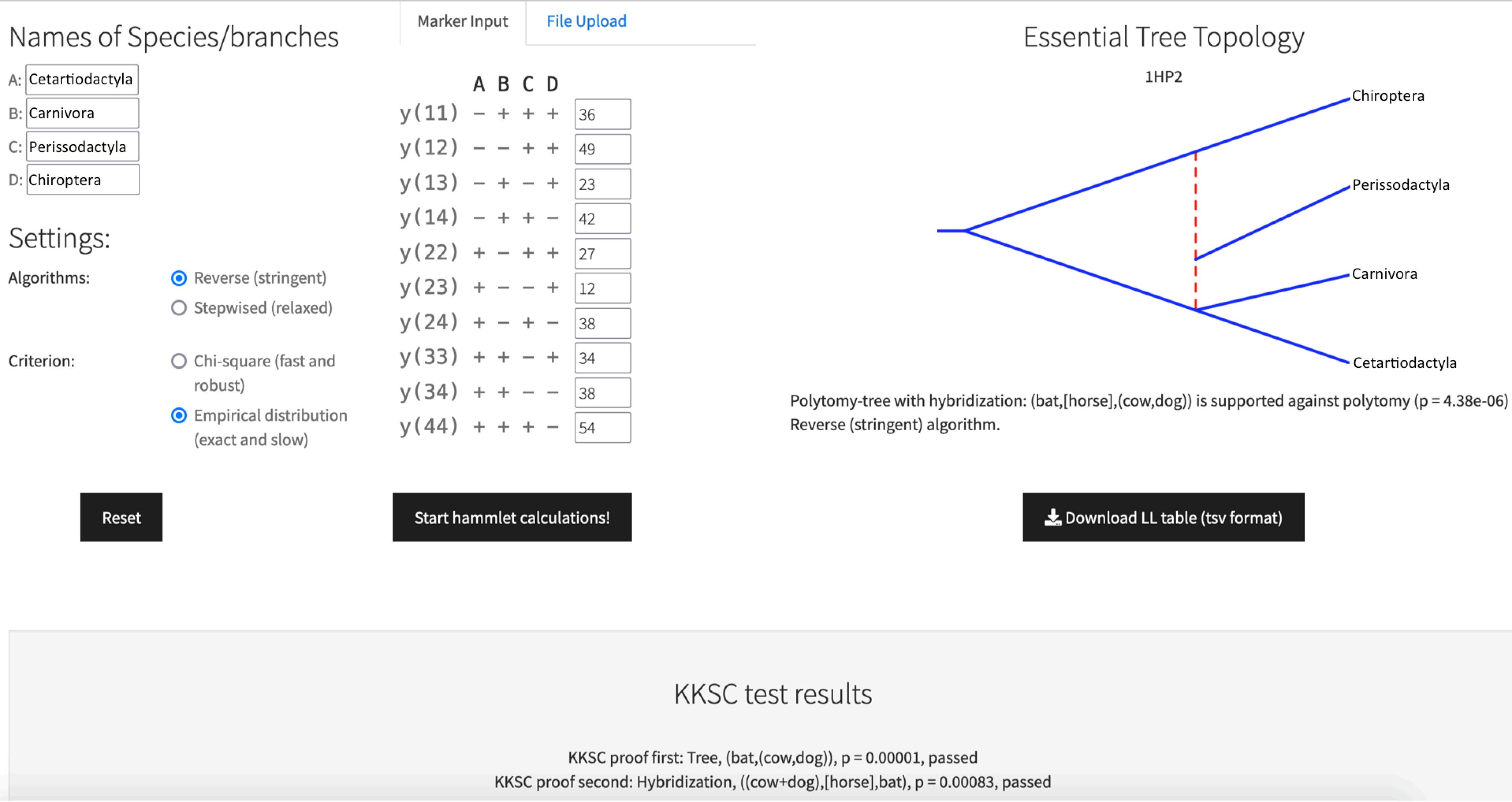

Supplement: Supplementary file 1 [file genes-13-00766-s001.zip › Supplementary_Materials/Supplementary_Figure_S2.pdf]
